# Combination of MRI-based prediction and CRISPR/Cas12a-based detection for IDH genotyping in glioma

**DOI:** 10.1038/s41698-024-00632-8

**Published:** 2024-07-01

**Authors:** Donghu Yu, Qisheng Zhong, Yilei Xiao, Zhebin Feng, Feng Tang, Shiyu Feng, Yuxiang Cai, Yutong Gao, Tian Lan, Mingjun Li, Fuhua Yu, Zefen Wang, Xu Gao, Zhiqiang Li

**Affiliations:** 1 Brain Glioma Center & Department of Neurosurgery, Zhongnan Hospital of Wuhan University, Wuhan, China; 2 Department of Neurosurgery, 960 Hospital of PLA, Jinan, Shandong China; 3 Department of Neurosurgery, Liaocheng People’s Hospital, Liaocheng, China; 4 Department of Neurosurgery, PLA General Hospital, Beijing, China; 5 Department of Pathology, Zhongnan Hospital of Wuhan University, Wuhan, China; 6 Department of Prosthodontics, Wuhan University Hospital of Stomatology, Wuhan, China; 7 Department of Radiology, Liaocheng People’s Hospital, Liaocheng, China; 8 Department of Physiology, Wuhan University School of Basic Medical Sciences, Wuhan, China; 9 Department of Neurosurgery, General Hospital of Northern Theater Command, Shenyang, China

**Keywords:** CNS cancer, Mathematics and computing

## Abstract

Early identification of IDH mutation status is of great significance in clinical therapeutic decision-making in the treatment of glioma. We demonstrate a technological solution to improve the accuracy and reliability of IDH mutation detection by combining MRI-based prediction and a CRISPR-based automatic integrated gene detection system (AIGS). A model was constructed to predict the IDH mutation status using whole slices in MRI scans with a Transformer neural network, and the predictive model achieved accuracies of 0.93, 0.87, and 0.84 using the internal and two external test sets, respectively. Additionally, CRISPR/Cas12a-based AIGS was constructed, and AIGS achieved 100% diagnostic accuracy in terms of IDH detection using both frozen tissue and FFPE samples in one hour. Moreover, the feature attribution of our predictive model was assessed using GradCAM, and the highest correlations with tumor cell percentages in enhancing and IDH-wildtype gliomas were found to have GradCAM importance (0.65 and 0.5, respectively). This MRI-based predictive model could, therefore, guide biopsy for tumor-enriched, which would ensure the veracity and stability of the rapid detection results. The combination of our predictive model and AIGS improved the early determination of IDH mutation status in glioma patients. This combined system of MRI-based prediction and CRISPR/Cas12a-based detection can be used to guide biopsy, resection, and radiation for glioma patients to improve patient outcomes.

## Introduction

Glioma is the most frequent primary tumor of the central nervous system, exhibiting a devastating prognosis^1^. Molecular classification contributes to a better understanding of glioma pathophysiology and disease stratification^2^. Isocitrate dehydrogenase (IDH) mutation status has emerged as one of the most important molecular markers for glioma diagnosis and therapy, and the early determination of IDH mutation status directly impacts treatment decisions^3,4^. Traditional detection methods based on immunohistochemistry (IHC) and next-generation sequencing (NGS) are time-consuming and are mostly used for postoperative diagnosis, which cannot meet the needs of early determination of IDH mutation status. The application of deep machine learning using radiomics images has shown potential to be used for the prediction of IDH mutation status^5–7^. Moreover, newer detection systems are continually being developed, some of which can rapidly provide molecular information during surgery^8,9^.

Magnetic resonance imaging (MRI) plays a leading role in non-invasive glioma diagnosis and treatment planning. Vast efforts have been devoted to preoperatively determining IDH mutation status using MRI radiographic features via deep learning algorithms^5,6,10–12^. Several approaches are used to develop multitask models for both segmentation and classification, for which segmentation masks are used for segmentation tasks^5,6,12^. However, automated tumor segmentation algorithms require manually segmented tumor images as training examples, and manually segmenting gliomas in MRI is a time-consuming task. In practice, clinically useful models are often costly, generally because of the burden of lesion segmentations from hundreds or thousands of medical images. Previous studies focused on the MRI textural features in glioma regions and ignored the information in these images on other parts of the brain, which have also been shown to be associated with IDH status^13,14^. IDH status affects the interaction between cells, and the interactive effects may occur in other parts aside from the lesion. For example, cells expressing IDH1 mutant release d-2-hydroxyglutarate (D-2HG), a product increasing neuronal activity by mimicking the activity of glutamate^15^, and the accumulated D-2HG in the circulating CSF made all neurons exposed to relatively high concentrations of D-2HG^16^. Besides, the connectome-based approach further revealed that the brain connectivity disruption, distant from the focal lesion, was different based on IDH status^17,18^. We hypothesized that extracting the imaging features from whole slices in MRI scans would improve the prediction accuracy of a classification model. In order to handle the complex image information from whole slices in brain MRI scans, Transformer, a novel deep learning architecture^19^, was utilized in this study. Transformer can effectively capture long-range contextual relations between image pixels while maintaining low-level feature extraction, and its noise suppression capabilities allowed Transformer to be used for more complex pattern recognition^19,20^.

Sensitive and rapid detection of gene mutation is essential for precision medicine, but detection tools with these attributes are still not widespread. In recent years, Clustered Regularly Interspaced Short Palindromic Repeats (CRISPR) systems have been utilized for rapid and sensitive nucleic acid detection because Cas proteins can accurately recognize nucleic acid targets of interest^21–23^. Cas12a is one of the most commonly used CRISPR/Cas proteins in DNA detection systems. Upon recognition and cleavage of target double-strand DNA, the collateral cleavage activity of Cas12a is activated, resulting in cleavage of nearby single-strand DNA (including fluorescence reporters) in a non-specific manner, and this “collateral cleavage” has been widely exploited for sensitive and specific detection of target sequences^24,25^. Here, we developed an IDH mutation detection instrument, which we have termed the CRISPR/Cas12a-based automatic integrated gene detection system (AIGS). In this tool, the combination of a rapid PCR amplification system, CRISPR/Cas12a-mediated cleavage assay, and real-time fluorescence quantification system have been used to realize fully integrated detection from sampling into a report.

The veracity and stability of gene testing results depend critically on accurate biopsy sampling from the tumor-rich tissues. However, obtaining tumor-rich tissue samples can often be a challenge. A report from The Cancer Genome Atlas (TCGA) suggests that only 35% of biopsy samples contain sufficient tumor content for appropriate molecular characterization^26^. Deep machine learning efforts with radiomics analysis have been used to extract numerous features in other cases to visualize spatial histologic heterogeneity and delineate biopsy targets in glioma. Several outstanding studies have shown proof-of-concept that MRI imaging and machine learning techniques can make clinically useful quantitative predictions of important histopathologic findings, such as tumor infiltration^27^, metabolic status^28^, and proliferation indices^29^. We hypothesized that our predictive model with high accuracy also correlated with certain pathological changes in glioma patients, and the non-invasive correlation of histology developed using this model may facilitate image-guided biopsy.

**Fig. 1:**
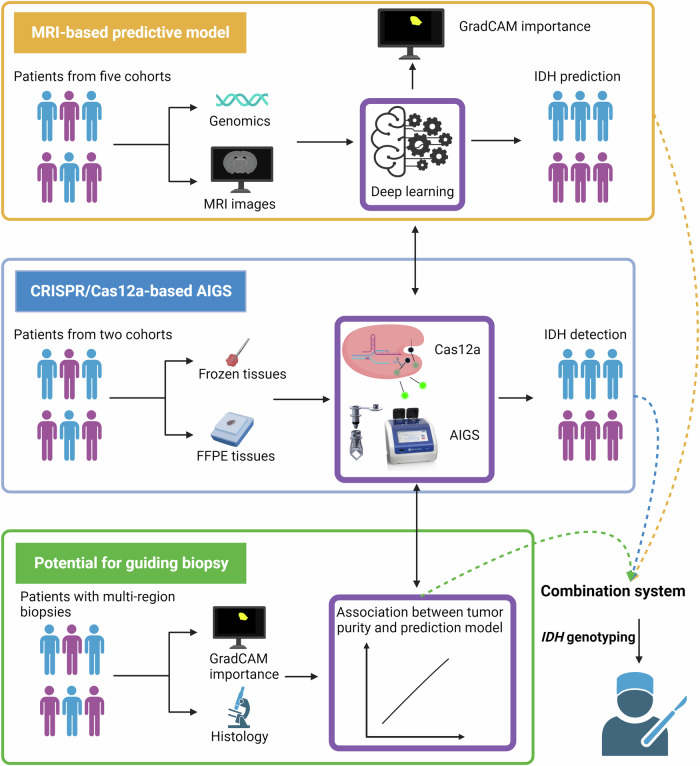
Graphical illustration of this study. The illustration was created with BioRender.com.

In this study, the objectives were threefold: (1) to establish an MRI-based predictive model for determining IDH mutation status using Transformer; (2) to develop CRISPR/Cas12a-based AIGS; and (3) to explore the clinical benefits of the combination of a predictive model and AIGS (details shown in Fig. 1).

## Results

### Patient characteristics

A total of 664 patients from five retrospective cohorts were used for constructing our predictive model (Supplementary Fig. S1). Patient cohorts from Zhongnan Hospital (ZNH, *n* = 261), General Hospital of Northern Theater Command (NTCGH, *n* = 121), and Liaocheng Peoples Hospital (LPH, *n* = 104) were used for model development and in internal test sets, and the cohorts from Chinese PLA General Hospital (PLAGH, *n* = 30) and The Cancer Imaging Archive (TCIA, *n* = 148) were used as external test sets. The prevalences of IDH-mutants in the development, internal test, PLAGH test, and TCIA test sets were 48.18%, 43.84%, 33.33%, and 38.51%, respectively. IDH status of these patients is shown in Supplementary Table 1. There was no significant difference for age (*p* = 0.30), sex (*p* = 0.56), IDH status (*p* = 0.12), or grade (*p* = 0.49) between these sets, but the prevalence of 1p/19q co-deletion was significant (*p* = 0.02) in the development set (Supplementary Table 2). Frozen tissues in the NTCGH cohort (*n* = 20) and FFPE tissues in the LPH cohort (*n* = 91) were used for evaluating the performance of CRISPR-based AIGS, and clinicopathological information for retrospective samples is shown in Supplementary Table 3. Glioblastoma accounted for the highest proportion, followed by astrocytoma and oligodendroglioma. Moreover, thirteen patients in ZNH, with 2–6 biopsies each, were recruited to explore the association between tumor cell percentage and the resulting feature attribution maps by prediction model (clinical data is given in Supplementary Table 4).

### MRI-based prediction model performance

**Fig. 2:**
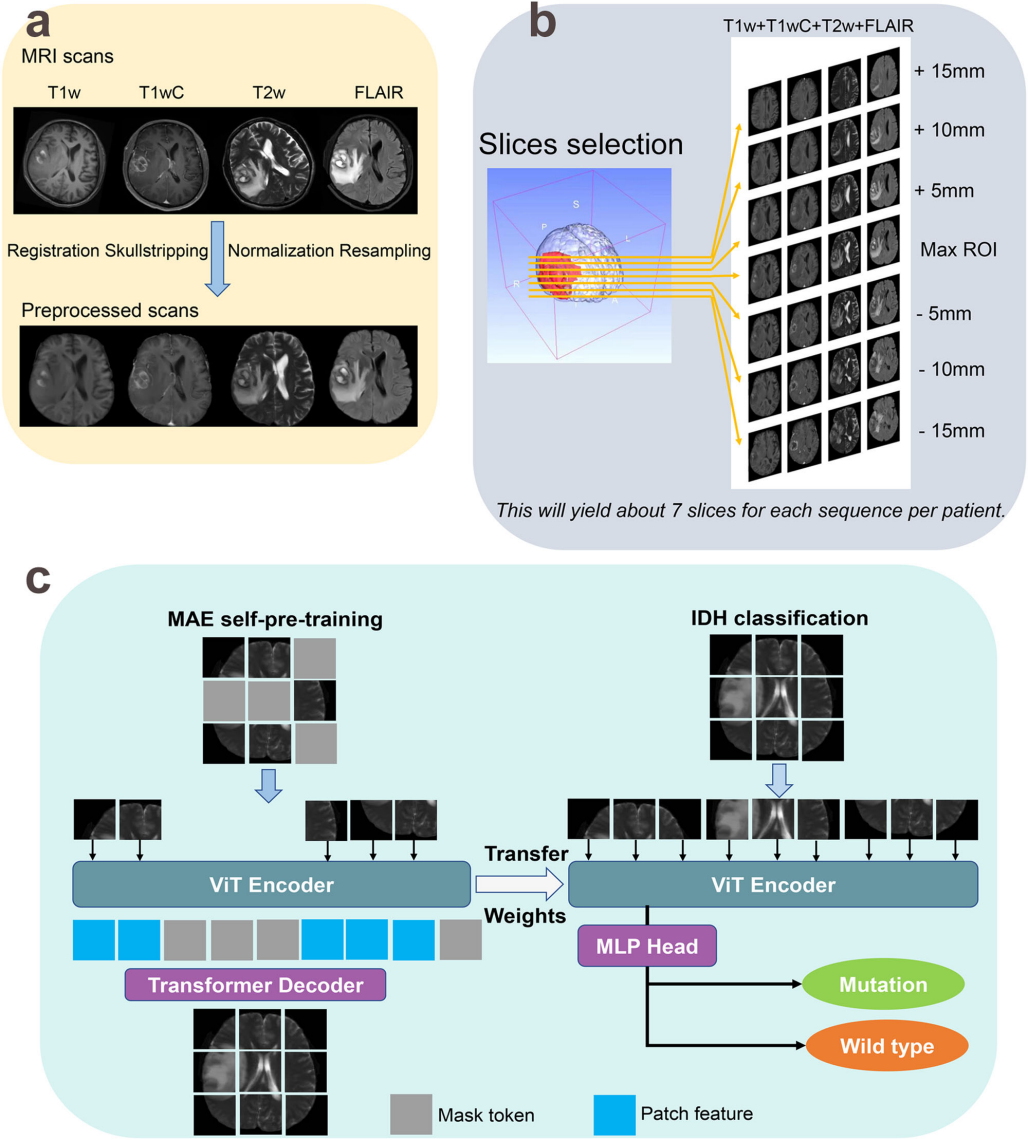
Construction of a predictive model of IDH status. **a** T1wC, T1w, T2w, and FLAIR scans were used as inputs, and these scans were registered to an atlas, skull stripped, normalized, and resampled. **b** Segmented FLAIR lesions were used to select slices by first selecting the central slice with the maximum glioma area and expanding every 5 mm until the boundary of each lesion was reached. **c** Classification pipeline with MAE self-pre-training. Left: A ViT encoder was first pre-trained with MAE. A random subset of patches was input to the encoder, and a transformer decoder reconstructed each full image. Right: Self-pre-trained ViT weights were transferred to initialize the segmentation encoder and a linear classifier was used for classification tasks.

The MRI data from all included patients were acquired in DICOM format, then the MRI scans were pre-processed (including registration to an atlas, skull-stripping, intensity normalization, and resampling) (Fig. 2a). To extract comprehensive signal intensity information from each tumor, masks for each segmented lesion volume were used to select the image slice containing the largest tumor area in the axial plane. Additional slices spaced 5 mm apart were added until the edge of the tumor mask was reached (Fig. 2b), and the axial slices per patient were automatically selected based on tumor segmentation and were considered individual samples for model development and testing. These slices were individually fed into the model, and the model was trained to classify 2D slices. We hypothesized that the IDH status of multiple 2D slices from a single subject could present the subject’s whole tumor state, and the diagnostic accuracy per patient was calculated from the mean value of the predicted probabilities for slices.

Vision transformers (ViTs)^20,30^ were used to predict the genetic features from MRI scans. Self-pre-training with masked autoencoders (MAE)^31^ was first performed, and another ViT with a task-specific head was used as the backbone network for constructing our classification model (Fig. 2c). The reconstruction results from MAE demonstrated that self-pre-training was able to restore lost information from a random context (Supplementary Fig. S2), which was conducive to the completion of classification tasks. The ablation study of MAE pretraining on mask ratios and epochs further proved its effect (Supplementary Table 5). The final model was achieved through 63 epochs of training, and the performance of this predictive model on test sets is summarized in Table 1. With mean probabilities from 7 samples per patient, our model achieved accuracies of 0.93, 0.87, and 0.84, with AUCs of 0.95 (95% CI: 0.93–0.97), 0.91 (95% CI: 0.87–0.94), and 0.89 (95% CI: 0.85–0.94) using the internal test, PLAGH, and TCIA sets, respectively. Besides, the prediction status for each IDH1/2 variant in the predictive model is shown in Supplementary Table 6.

**Table 1:** Diagnostic performance of the model for the prediction of IDH status

Dataset	AUC (95% CI)	Accuracy	Sensitivity	Specificity
*Per slice*				
Internal test set	0.93 (0.91–0.95)	0.90	0.88	0.92
PLAGH set	0.90 (0.93–0.87)	0.86	0.77	0.90
TCIA set	0.86 (0.82–0.90)	0.82	0.77	0.95
*Per patient*^*a*^				
Internal test set	0.95 (0.93–0.97)	0.93	0.91	0.95
PLAGH set	0.91 (0.87–0.94)	0.87	0.90	0.85
TCIA set	0.89 (0.85–0.94)	0.84	0.77	0.88

AUC, area under the receiver operating characteristic curve.

^a^Since each patient yielded about 7 tumor slices, the diagnostic accuracy per patient was calculated from the mean value of the predicted probabilities for slices.

To provide insight into the behavior of this model, GradCAM images were created, and the selected filter outputs from this established model were visualized. These maps and visualizations showed which parts of each scan contributed the most to each prediction (Supplementary Fig. S3). For patients with correct predictions, the model focused on the tumor region, whereas, for patients with incorrect predictions, the model failed to do this. The filter output visualizations recognized specific imaging features such as the T2-weighted (T2w) brightness. The ring-enhancing lesion surrounding a necrotic core in the T1-weighted contrast-enhanced (T1wC) scan seemed to be the feature that contributed to a correct prediction for IDH-wildtype gliomas. The absence of enhancement or minimal enhancement in the T1wC scan was the feature of tumors with IDH mutations, which was consistent with the previous studies^32–34^.

### Development of a one-pot CRISPR/Cas12a-based assay

**Fig. 3:**
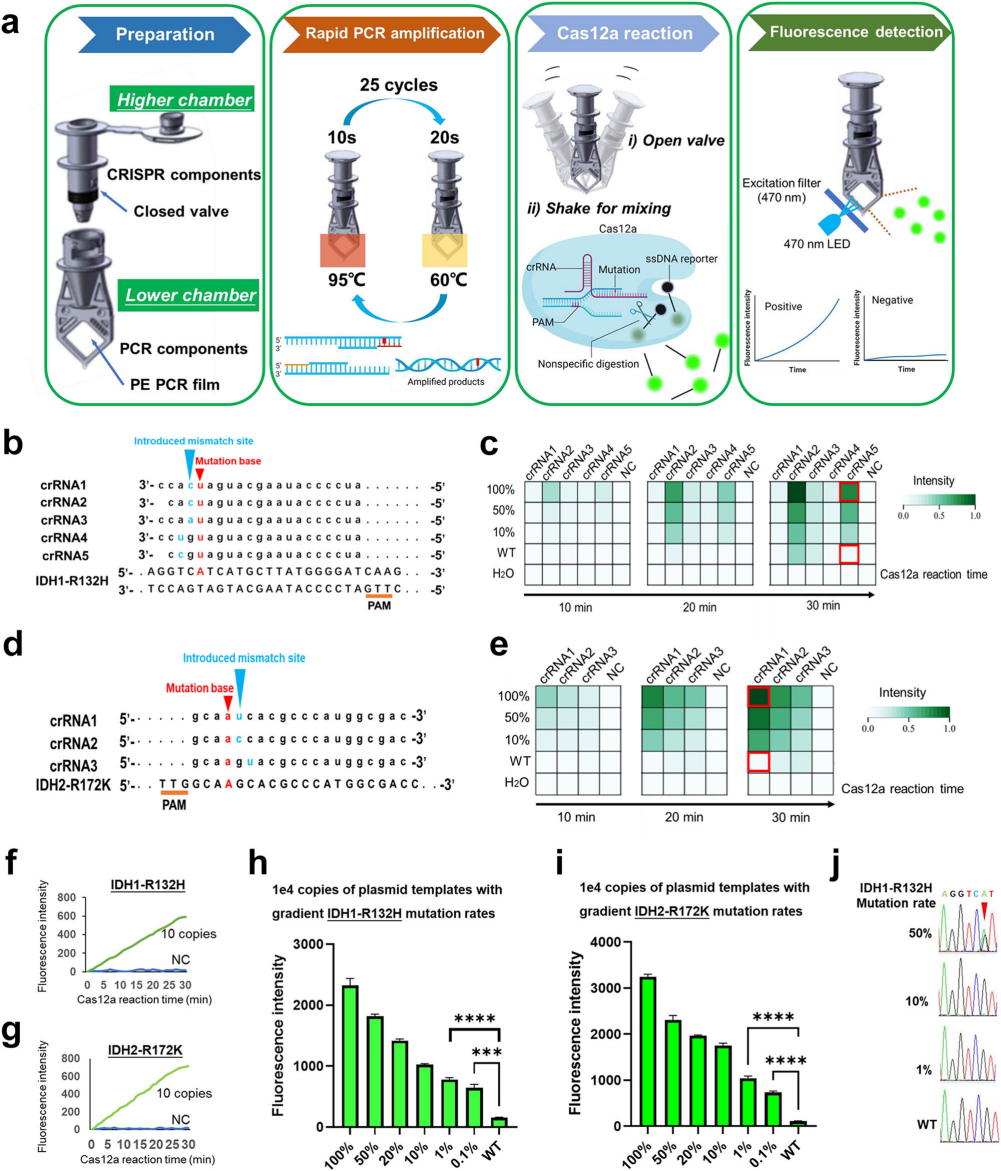
Development of a one-pot CRISPR/Cas12a-based assay. **a** Schematic of a one-pot method for IDH mutation status detection. Sequences of **b** IDH1-R132H and **d** IDH2-R172K crRNAs. Fluorescence heatmaps of different **c** IDH1-R132H and **e** IDH2-R172K crRNAs in Cas12a reactions. Time-course analysis of detecting 10 copies of **f** IDH1-R132H and **g** IDH2-R172K templates using our one-pot assay. Sensitivity of CRISPR/Cas12a-based detection of 1 × 10^4^ templates with a gradient of **h** IDH1-R132H and **i** IDH2-R172K mutation rates. Data are presented as the mean ± SD (*n* = 3). ***p < 0.001, ****p < 0.0001 versus the WT group. **j** Sanger sequencing chromatograms of mutant dilutions. Minor components of the figure were obtained from BioRender.com.

Mutation detection based on CRISPR/Cas12a is rapid and sensitive, and a combination of this method with rapid PCR can make it faster and more accurate. For integrating rapid PCR amplification and Cas12a cleavage into a one-pot reaction system, we designed a specific reaction tube, which greatly simplified the operation and avoided amplicon contamination when detecting IDH mutation. There was a controllable valve in the middle of the reaction tube, separating the reaction tube into two chambers. The lower chamber is composed of PE PCR film, allowing for better thermal transfer to promote the efficiency of PCR, and rapid PCR reagents were deposited on this side of the vessel. The reagents of our Cas12a cleavage system were stored in the higher chamber of the tube. When the amplification is complete, the CRISPR/Cas12a-mediated cleavage system is then mixed into the lower chamber by opening the valve and shaking the tube. Following the completion of the cleavage reaction, the fluorescence of each sample was then measured (Fig. 3a).

To identify the optimal CRISPR RNA (crRNA) for detecting IDH1-R132H, five crRNAs were designed (Fig. 3b). We compared the sensitivity and specificity of these crRNAs in detecting IDH1-R132H in templets at 1 × 10^10^ copies, comprising the R132H and WT alleles. After 30 min of a Cas12a reaction, all crRNAs, particularly crRNA2, and crRNA5, could detect each sample with 100% D835Y, while crRNA1 but not crRNA5 produced a strong signal even for the WT allele (Fig. 3c). Thus, crRNA5 was the optimal crRNA for IDH1-R132H detection based on its excellent sensitivity and specificity. An optimal crRNA for IDH2-R172K was similarly identified (Fig. 3d, e). Next, eight pairs of rapid PCR primers were screened to improve sensitivity (Supplementary Fig. S4). Using the best primer pairs and crRNAs, as low as 10 copies of IDH1-R132H or IDH2-R172K templates could be detected after 30 min of rapid PCR and 30 min of Cas12a reaction (Fig. 3f, g). Moreover, the detection limit for the system was determined to be 0.1% with 1 × 10^4^ copies of the template as an input (i.e., 10 copies of IDH1-R132H template mixed with 9990 copies of WT template) (Fig. 3h). A similar detection limit for IDH2-R172K was also determined (Fig. 3i). We next compared this one-pot system with a commonly used Sanger sequencing method. Sample Sanger sequencing chromatograms of mutant dilutions show that at 10%, the mutation was not reliably detectable over the background (Fig. 3j).

### Construction of a CRISPR/Cas12a-based detection device

**Fig. 4:**
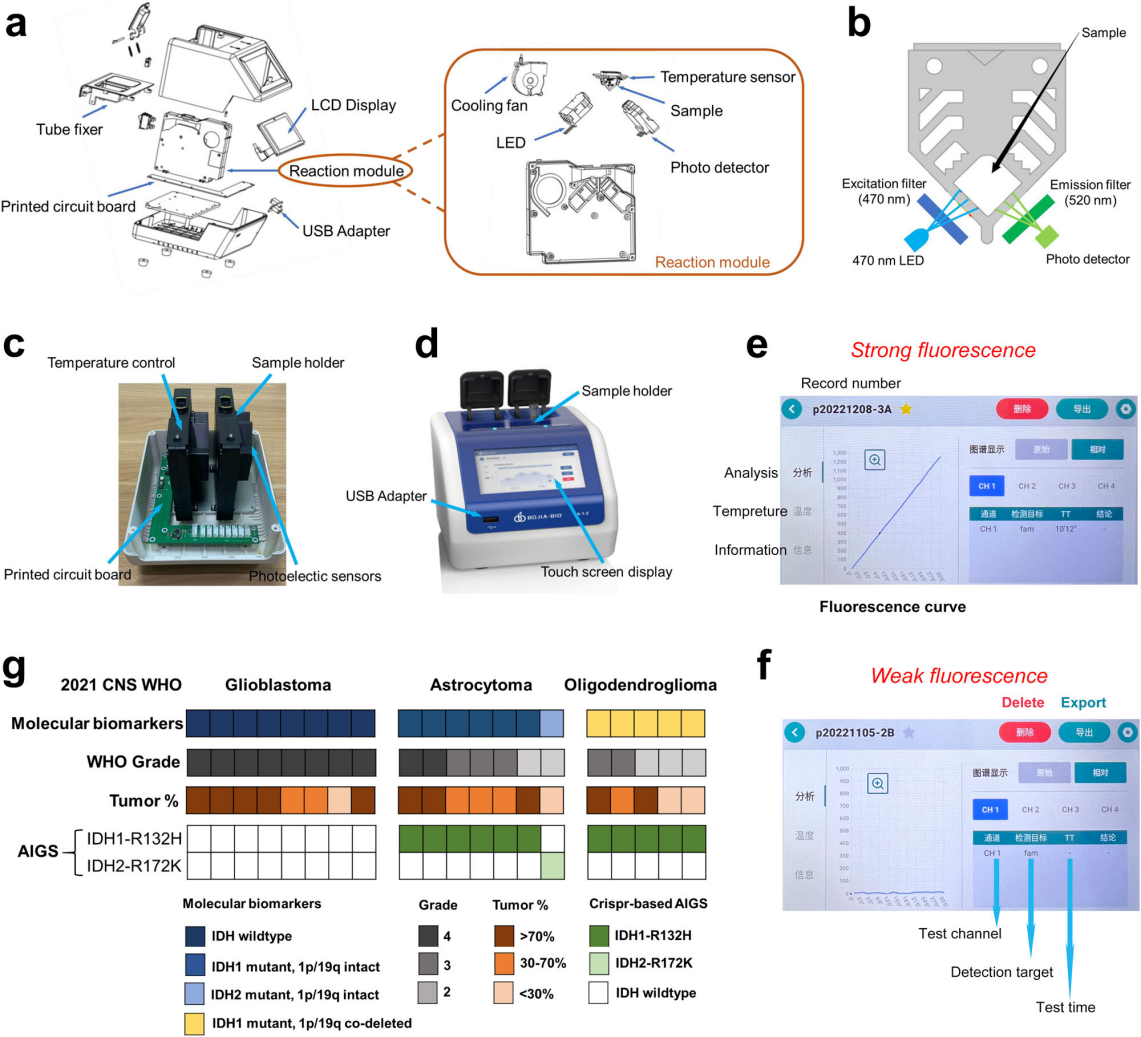
Construction of CRISPR/Cas12a-based AIGS. **a** Simplified schematic diagram of the optical system, heating system, and fluorescence detection subsystems of the AIGS system. **b** Schematic diagram of the optical path in the optical system. **c** Internal structure of the instrument. **d** Images of the AIGS system. **e** A typical detection result from AIGS. The results can be observed by monitoring fluorescence curves. **f** Test results using negative samples under the same conditions. **g** A cohort of frozen tumor samples was assayed for IDH mutation by AIGS (*n* = 20).

To facilitate the promotion and commercialization of this one-pot CRISPR/Cas12a-based assay, we designed and constructed an automatic integrated gene detection system (AIGS). The core components and optical paths of this AIGS are shown in (Fig. 4a, b). In brief, the device mainly includes an optical system, a temperature control system, and a timing control system. In the optical system, a 470 nm light-emitting diode (LED) was used as the fluorescence excitation module, and a photodetector was mounted on the other side of the reaction tube. The temperature control module was located at an orientation to control the optimal temperature for rapid PCR and CRISPR/Cas12a-mediated cleavage, and the timing control was a single-board computer. We used three-dimensional (3D) printing technology to prepare all brackets and shells for the fixing and assembly of these components (Fig. 4c). To simplify the operation, we designed a touchscreen-based man–machine interface. After the reaction tube is put into the sample table and the START button is clicked, rapid PCR amplification (30 min) and CRISPR detection (30 min) procedures (Fig. 4d) are performed automatically. In AIGS, real-time results of fluorescence curve changes are displayed on the touchscreen panel. The display interface from a typical detection result is shown in Fig. 4e, and the corresponding negative control test results are shown in Fig. 4f. Additionally, another device with eight detection channels was designed for multiplex molecular detection (Supplementary Fig. S5).

We assayed a cohort of frozen tissue specimens (*n* = 20) representing the major subtypes of diffuse glioma by AIGS, including varying WHO grades (2–4), histologies (GBM, astrocytoma, and oligodendroglioma), and highly variable tumor percentage (3–100%) (Fig. 4g). AIGS achieved 100% diagnostic accuracy for IDH detection. A cohort of glioma FFPE cases (*n* = 91) was then tested, and AIGS gave identical results to available original clinical molecular marker results (Supplementary Table 7).

### Association between tumor purity and predictive model

A group of patients with newly diagnosed glioma was recruited from ZNH (4 diffuse astrocytomas, IDH mutant; 2 oligodendroglioma, IDH mutant and 1p/19q codeleted; 5 glioblastoma IDH wildtype). Based on conventional imaging findings in these MRI scans, two to six sphere targets of 5 mm in diameter were defined as biopsy targets for each patient. Two expert neuropathologists, blinded to the imaging results and patient diagnosis, independently assessed the percentage of cancer cells in each sample. For certain samples with IDH mutation, droplet digital PCR (ddPCR) was used to quantify the fractional abundance of the IDH1-R132H mutation. Whole exome sequencing (WES) was also performed on the tumor tissue samples without IDH mutation, and tumor purity for each sample was estimated from our WES data using the ABSOLUTE algorithm^35^.

**Fig. 5:**
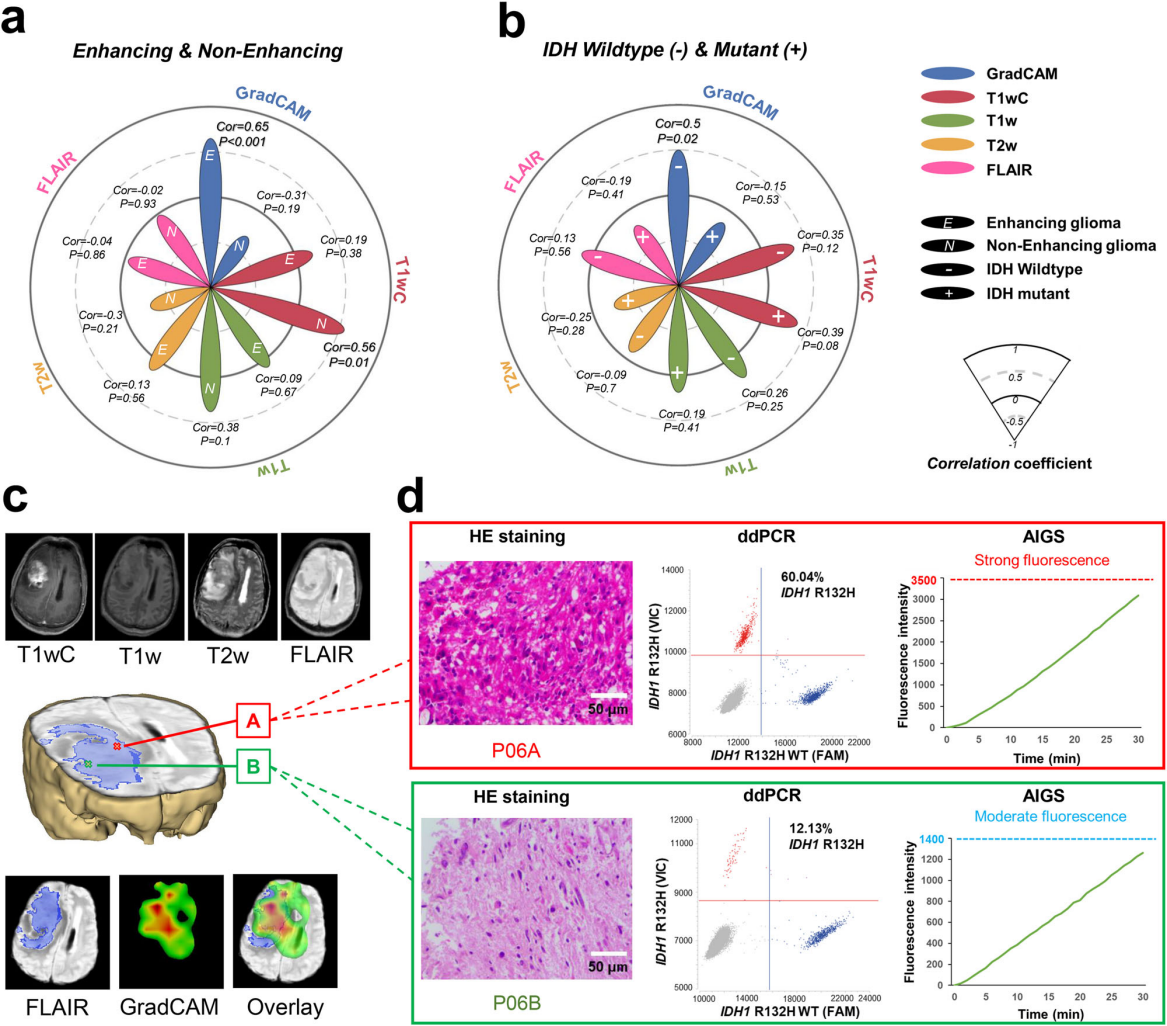
The potential of GradCAM heatmaps for guiding biopsy. Correlations between image intensity and tumor percentage in **a** enhancing/non-enhancing and **b** IDH-wildtype/IDH-mutant gliomas. Lengths of the five flower petals are qualitatively based on correlation indices and indicate the coefficients relative to the four circles representing −0.5, 0, 0.5, and 1. **c** MRI images and GradCAM heat map for patient 06. **d** Corresponding HE and ddPCR results of MRI-guided biopsy targets (cross points).

The MRI scans from the above patients were used as input into our trained model to predict the status of IDH mutation, and the important image regions that influenced these predictions were identified by GradCAM. For all of the multiple biopsy samples, imaging measurements were not associated with glioma cell percentages (Supplementary Fig. S6). In subgroup analysis, the highest correlations with tumor cell percentage in enhancing and IDH-wildtype gliomas were found to have GradCAM importance (0.65 and 0.5) (Fig. 5a, b and Supplementary Fig. S7). A GradCAM heatmap for patient 06 (a 31-year-old male patient with an enhancing oligodendroglioma, IDH-mutant) was generated and is shown in Fig. 5c, and a raised tumor percentage was observed in the P06A region with its high GradCAM importance. Then ddPCR was used to further validate the presence of differences in tumor purity, and we found that the allele fraction (IDH1-R132H) in this important region was higher than that in unimportant regions (60.04% for P06A vs. 12.13% for P06B). Furthermore, the fluorescence intensity of the P06A sample was stronger than that of P06B at the same reaction time by AIGS (Fig. 5d), which suggested that using GradCAM heatmaps to guide biopsy could improve accuracy rates and shorten observation time when using AIGS.

**Fig. 6:**
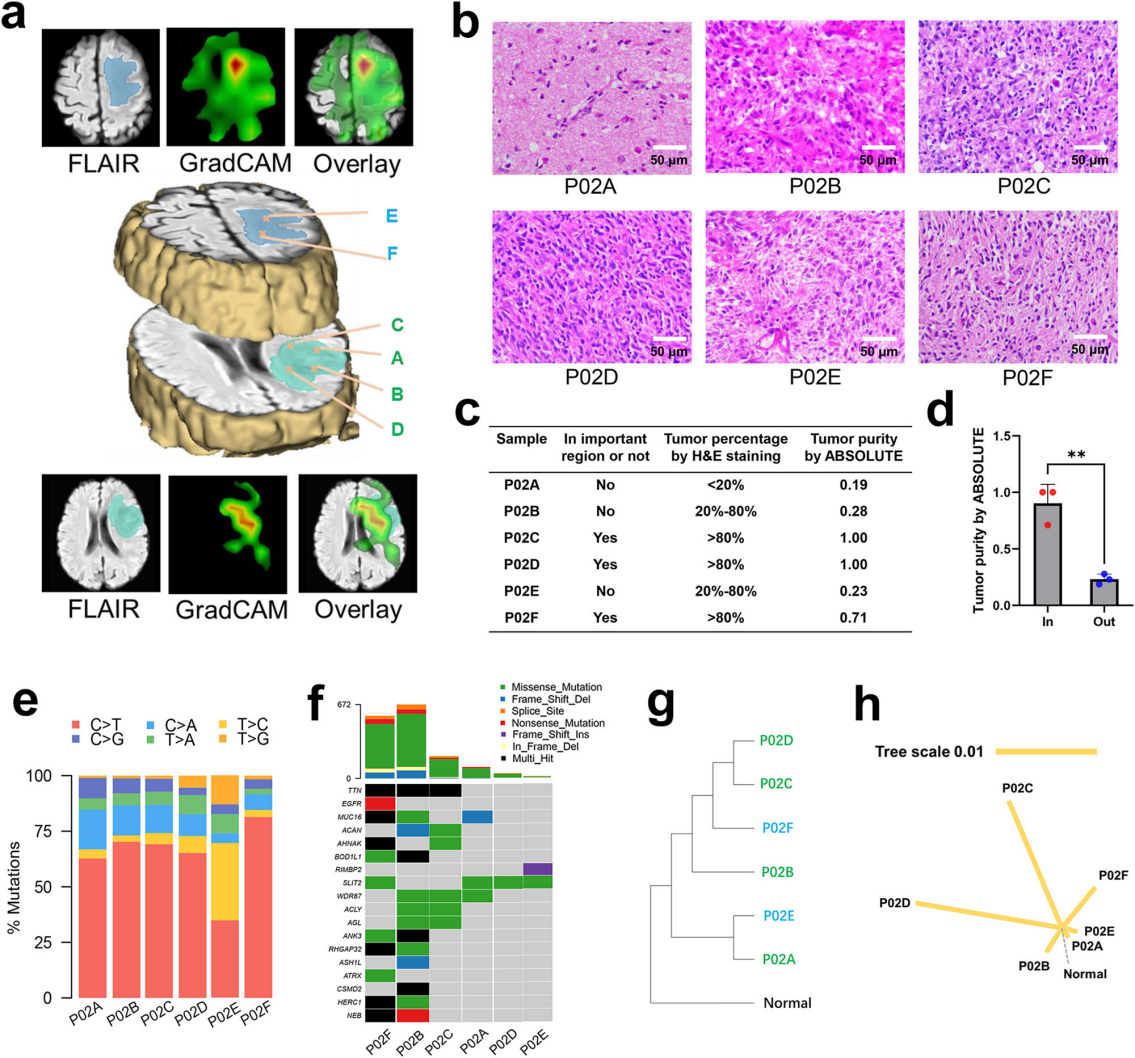
Example of imaging with corresponding histology. **a** MRI images and GradCAM heatmaps for patient 02. **b** Corresponding HE of biopsy targets. **c** Statistical table of tumor position, cancer cell percentage, and tumor purity of biopsy targets. **d** Comparison of tumor purity between important regions and unimportant regions as estimated by GradCAM. Data are presented as the mean ± SD (*n* = 3). **p < 0.01 between important regions and unimportant regions. **e** The frequ**e**ncy of base substitutions within specific trinucleotide mutational contexts of each sample in patient 02. **f** Oncoplot o**f** genes with highest counts of variants in multi-spot tumors. **g** Phylogenetic tree of patient 02 by iTOL. **h** Phylogenetic tree was constructed for patient 02 based on the maximum parsimony algorithm.

Another representative example with six biopsy targets (patient 02, a 71-year-old female patient with an enhancing glioblastoma, IDH wildtype) is shown in Fig. 6a. Consistent with the above, the tumor purities in the important regions (P02C, P02D, and P02F) were higher than those in the regions with less importance (P02A, P02B, and P02E), as measured by both HE and sequencing using the ABSOLUTE algorithm (Fig. 6b–d). Tumor heterogeneity was estimated by WES (Supplementary Fig. S8), and different frequencies of base substitutions and gene variants were observed in different samples from patient 02 (Fig. 6e, f). A phylogenetic tree was next constructed to depict clonal relationships and ordering events (Fig. 6g, h). The tree was barely rooted in patient 02, and the samples in important regions were more closely related in this tree, which indicated that our predictive model identified tumor heterogeneity.

Taken together, the correlations between tumor cell abundance and GradCAM importance in our predictive model were generated by the following observations. First, the higher estimated tumor purity was almost always inside and around the contrast-enhancing volume for enhancing tumors. This was consistent with clinically known proliferation-leakage associations. Second, the higher glioma cell percentage was almost always close to cisterns in IDH-wildtype gliomas, which might be related to the role of subventricular zone (SVZ) stem cells in glioblastoma^36–38^.

## Discussion

In this study, we combined a transformer-based neural network with MAE self-pre-training to identify genetic features from the entire brain rather than only within tumor areas present in T1wC or fluid-attenuated inversion recovery (FLAIR), and the accuracy of this predictive model was as high as 0.84–0.87 using external test datasets. Besides, GradCAM analysis was also performed to explore the behavior of our model, and T2w brightness and ring-enhancing lesions in T1wC scans seemed to be the outstanding features extracted by this model in enhancing gliomas. We were surprised to find that this model could focus on a region with high tumor purity in enhancing gliomas, and the correlation between GradCAM importance and cancer cell percentage reached as high as 0.65.

Most existing MRI-based IDH classification models depend on multitask models, for which segmented lesions in the segmentation model are used for further classification^39^. However, these models require a large number of manually labeled images for training, and manual segmentation at the voxel level is time-consuming and costly. This motivated us to develop models without relying on lesion segmentation. Building a predictive model of IDH status directly from whole slices in MRI scans is more challenging than deriving a model using segmented lesions because the most area is heathy tissue that might be unrelated to IDH status. However, this decreased association does not implicitly indicate that these tissues have no association with IDH status. Many lines of evidence, at both the molecular and cellular levels, have demonstrated that glioma is a whole-brain disease^13,40,41^. Three factors contributed to the transformer’s superiority for feature learning in this study: (1) Multi-head self-attention derived from the transformer’s good noise suppression ability. Specifically, due to the inherent heterogeneous nature of glioma tumors and lesion boundary diffusion, quite a lot of noise can be mixed with information related to IDH genotyping; (2) Shifted windows ensure global information interactions. Transformer can effectively capture long-range contextual relations between image pixels while maintaining low-level feature extraction capabilities; (3) Transformer has robust adaptability. Fine-tuning pretrained Transformer enables a model to adapt to and perform better on downstream tasks^19,42^. One aspect that has garnered recent attention is the management of low-grade gliomas that are incidentally detected without objective clinical signs during MRI screening for unrelated syndromes. Emerging evidence emphasizes the importance of pursuing early interventions to achieve maximal recovery, potentially prolonging survival and delaying malignant progression in small “incident” gliomas^43,44^. However, we could not accurately conclude whether this MRI model has good accuracy for smaller “sporadic” gliomas, as all the enrolled patients had obvious clinical signs. Ensuring the predictive ability of the model for IDH in small “incident” gliomas and distinguishing them from high-grade gliomas in a timely manner will aid with the surgical approach of these patients. Therefore, further research remains necessary.

Traditional approaches for mutation detection, such as Sanger sequencing, lack sensitivity or speed, and accuracy is often seriously affected by heterogeneity and low tumor purity^45,46^. Based on the mechanism of *trans* cleavage by Cas12a, CRISPR/Cas12a systems have recently been shown to have great potential for molecular diagnostics^47–50^. Currently, we have only designed mutation detectors for IDH1-R132H and IDH2-R172K variants. In gliomas, R132H is the most common IDH1 mutation, and R172K is the most common IDH2 mutation^51,52^. Further judgment was still required when the AIGS report identified a negative result. However, considering that IDH1-R132H and IDH2-R172K are the most common variants if the two variants specified were not detected for tumors, we still encourage doctors to regard them as IDH wildtype gliomas and perform clinical treatment, especially when immediate judgment is required. If there is sufficient time, NGS should be performed to further improve the results obtained by AIGS. Besides, more detection markers of molecular characteristics, such as promoter methylation of MGMT, need to be developed to improve the range of IDH mutation status assays in clinical applications. Intraoperative rapid molecular diagnosis is important for aiding a neurosurgeon in surgical approaches, and the AIGS system established here with eight channels makes this possible at some future date (Supplementary Fig. S4). To further optimize AIGS, some measures need to be taken to reduce the detection time to approximately 30 min, such as using graphene to accelerate heat conduction for rapid PCR.

**Fig. 7:**
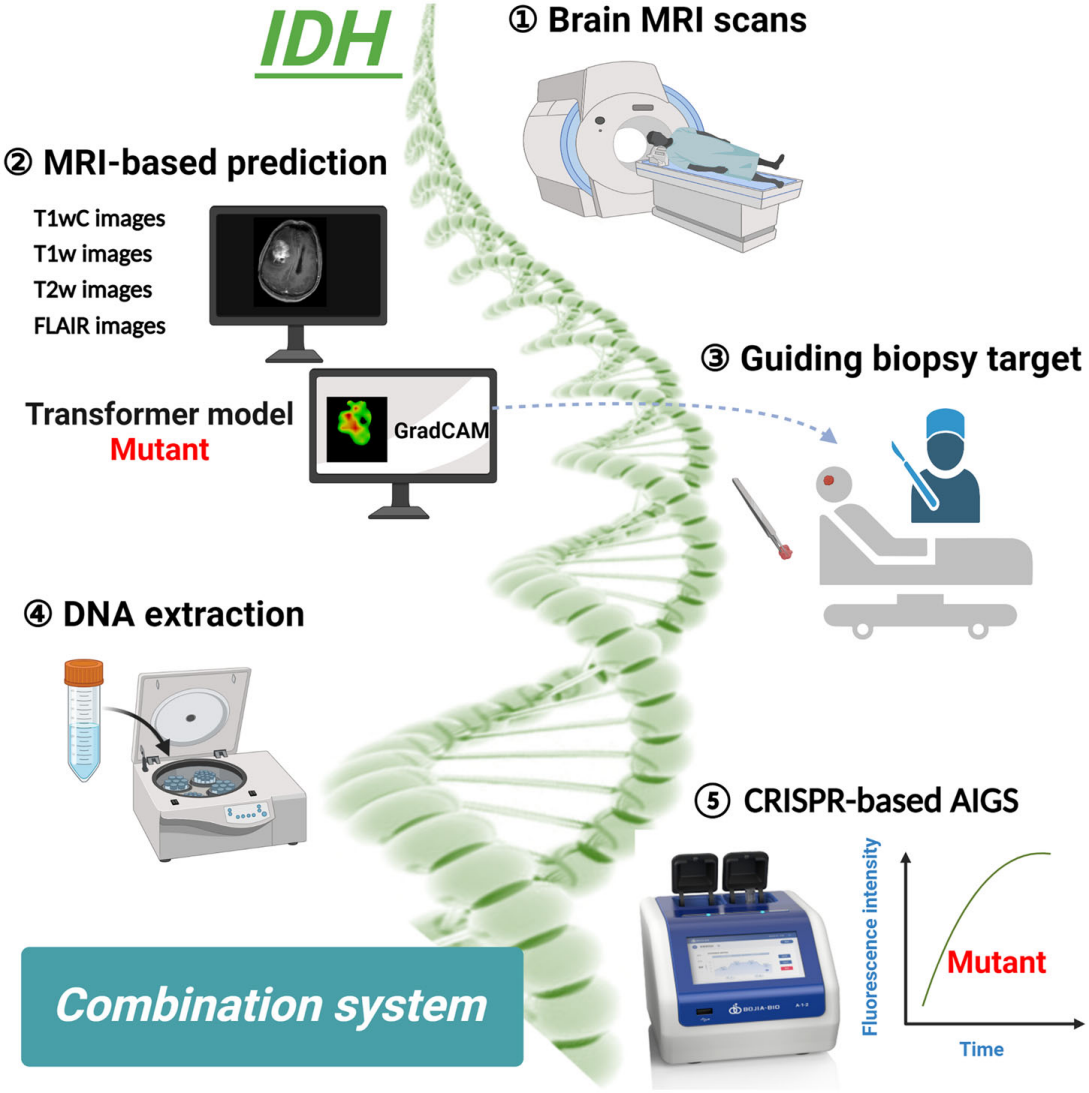
Combined MRI-based predictive and CRISPR/Cas12a-based AIGS system. Minor components of the figure were obtained from BioRender.com.

As for improving the diagnostic accuracy of AIGS, a tumor-rich sample being collected initially is very helpful. Some studies have suggested that MRI imaging has a certain facility for detecting glioma infiltration^27,53–55^. Verburg et al. reported that the diagnostic accuracy for tumor infiltration was highest for T1wC in non-enhancing gliomas^27^, which was similar to our findings. For our predictive model, the high correlation between extracted features and tumor cell percentage for enhancing gliomas indicated that the heat maps from GradCAM could be utilized to aid image-guided therapies. Therefore, we propose a combined system of preoperative prediction and intraoperative detection (Fig. 7), which should produce enhanced clinical benefits. In this combined system, machine learning with radiomics analysis could extract numerous features to visualize spatial histologic heterogeneity and delineate tumor-rich biopsy targets in glioma, and the non-invasive correlates of histology developed by this predictive model may facilitate image-guided biopsy to ensure the veracity and stability of the detection results provided by AIGS. This system could assist pathologists in making more accurate histological diagnoses, and it might satisfy clinical needs for determining clinical trial eligibility for trials studying neoadjuvant or intratumoral targeted drugs^56^. Although a limitation of our study is that we were not able to verify the effectiveness of this system using a prospective randomized controlled trial, the promise of the combination of a predictive model and rapid detection is still clear from the results presented herein and represents a valuable contribution to the scientific community. To the best of our knowledge, this is the first work that shows how to integrate a predictive model and rapid detection method for gene mutation.

In conclusion, we developed an MRI-based predictive model and CRISPR-based AIGS for IDH mutation status analysis. The feature importance identified by our predictive model can be represented using heat maps for biopsy, which can enhance the veracity and reliability of any detection result provided by AIGS. This combined system utilizing a predictive model and AIGS has great potential to accelerate precision medicine in the treatment of glioma.

## Methods

### Patient selection

A total of 1109 patients from five retrospective cohorts were considered for inclusion to construct this predictive model. Requirements for informed consent were waived, as no protected health information was reported herein. The details on inclusion criteria are provided in Supplementary Methods.

For testing CRISPR/Cas12a-based AIGS, tumor samples, and basic information on patients were obtained from NTCGH and LPH. A cohort of frozen tissue specimens (*n* = 20) representing the major subtypes of diffuse glioma were assayed by AIGS. Glioma formalin-fixed, and paraffin-embedded (FFPE) tissues with limited DNA from LPH (*n* = 94) were further used to test the accuracy of AIGS. The requirements for informed consent were waived, as no protected health information was reported herein.

To explore the association between pathological characteristics and the recognized features identified using our predictive model, multi-region stereotactic biopsies were harvested from the recruited patients from ZNH from July 2021 to February 2022 (*n* = 15), and in this case, these patients provided written informed consent. The details of IDH status evaluation for all patients in this study are presented in Supplementary Methods. The study was conducted in accordance with the guidelines of the Declaration of Helsinki and was approved by the Ethics Committee of Zhongnan Hospital (approval No. 2019048).

### Predictive Model Construction

The processing steps for MRI scans and the details of the model construction are summarized in Supplementary Methods. The performance of this model was then evaluated on the independent test sets. The area under the receiver operating characteristic curve (AUC), accuracy, sensitivity, and specificity were all evaluated for each genetic feature. For the AUC, we also evaluated the 95% confidence interval (CI) using basic bootstrapping with 1000 iterations. The model explanation was performed using Gradient-weighted Class Activation Mapping (GradCAM), a heatmap-based feature attribution method^57^. This approach enables rapid visual verification of our models’ performance by extracting features from regions that correspond to human interpretation.

### CRISPR/Cas12a-based AIGS development

In the Cas12a reaction, a mutation-specific crRNA is necessary to avoid cross-reactivity with the WT. To find the optimal crRNA for detecting IDH1-R132H and IDH2-R172K, five crRNAs for IDH1-R132H and three crRNAs for IDH2-R172K with different mismatches were designed. The sensitivity and specificity of these crRNAs in detecting mutations in the mixture of R132H/R172K and WT templates were evaluated. Primer design for PCR amplification was done using Prime Premier 5.0. The rapid PCR amplification system consisted of 25 μL 2× Hieff® Robust PCR Master Mix (Yisheng Biotechnology), 10 μM forward primer, 10 μM reverse primer, 40–100 ng DNA, and sterile water up to 50 μL. Rapid PCR was performed with a hot start at 95 °C for 180 s, followed by 25 cycles of [95 °C for 10 s and 60 °C for 20 s], respectively. Using the optimized crRNAs and primers, the lower limit of detection for the one-pot CRISPR/Cas12a-based assay was determined for IDH1-R132H and IDH2-R172K. The Cas12a-mediated cleavage system contained 1.5 μM Fncas12a, 3 μL 1× NEB buffer 2.1, 0.75 μM crRNA, 50 pM ssDNA FQ reporter, and 50 μL amplified products. The reaction was carried out at 37 °C for 30 min, with fluorescence collected every 1 min. The mutant and WT templates were mixed at various ratios, with IDH1-R132H or IDH2-R172K comprising 100%, 50%, 20%, 10%, 1%, and 0.1% of the total templates.

For facilitating the promotion and commercialization of this technology, we designed and constructed an automated integrated gene detection system (AIGS) (Supplementary methods). Three-dimensional (3D) printing technology was used to prepare all the brackets and shells for fixing and assembling these components, and a touchscreen-based man–machine interface was designed to simplify instrument operation. Frozen tissues and FFPE tissues of glioma were further used to test the reliability of AIGS.

### Tumor heterogeneity exploration

Imaging sequence coordinates that corresponded with biopsy sample locations were used to center cubic regions of interest (ROIs) of the 5-mm-diameter sphere. In order to normalize the imaging sequences with relative measurements (T1wC, T1w, T2w, FLAIR), an ROI was manually placed in the same region of the contralateral hemisphere for each biopsy location. For each imaging sequence, the mean of the voxel measurements within the biopsy and contralateral ROI were extracted for further analyses (ITK-SNAP software). In the heat maps generated by GradCAM, the intensity of the color was used to quantify the important region, and then Spearman’s correlation between GradCAM importance and tumor cell percentage was analyzed. The computation procedure of GradCAM importance is shown in Supplementary Methods. For certain samples with IDH mutation, droplet digital PCR (ddPCR) was used to quantify the fractional abundance of the IDH1-R132H mutation. Whole exome sequencing (WES) was also performed on the tumor tissue samples without IDH mutation, and tumor purity for each sample was estimated from our WES data using the ABSOLUTE algorithm^35^. The technical details of ddPCR and WES are given in Supplementary Methods. The correlation between tumor abundances and image intensities was calculated.

### Statistical analysis

Clinicopathological characteristics were compared using appropriate statistical tests, including Chi-square or Fisher’s exact test for categorical variables and t-test for continuous variables. The AUC, sensitivity, and specificity were used to evaluate the model classification ability. The Spearman’s correlation between GradCAM importance and tumor cell percentage was analyzed. All tests were two-sided, and statistical significance was defined as *P* < 0.05. Statistical analyses were performed using R software version 4.1.3.

### Supplementary information

Supplemental Material

## Data Availability

Requests to original datasets should be made directly via corresponding author (M.D., PhD, Zhiqiang Li, E-mail: lizhiqiang@whu.edu.cn) with a data access request form, institute rules and regulation of data access should be followed.

## Code availability

All code was implemented in Python 3.9 and R 4.1.3. Original Vit model code is available on https://github.com/google-research/vision_transformer. Original MAE model code is available on https://github.com/facebookresearch/mae. Custom Python and R code related to this study is publicly available on https://github.com/Tofu-fish/Vit-for-IDH-genotyping.
