# Supplementary material for: Combination of MRI-based prediction and CRISPR/Cas12a-based detection for IDH genotyping in glioma
Source: NPJ Precis Oncol. 2024 Jul 1;8:140. doi: 10.1038/s41698-024-00632-8 (PMC11217299; doi:10.1038/s41698-024-00632-8)
Supplement: Supplementary file 1 — Supplemental Material [file 41698_2024_632_MOESM1_ESM.pdf]

Supplementary Information

Combination of MRI-based prediction and CRISPR/Cas12a-based detection for IDH genotyping in glioma

Donghu Yu, Qisheng Zhong, Yilei Xiao, Zhebin Feng, Feng Tang, Shiyu Feng, Yuxiang Cai, Yutong Gao, Tian Lan, Mingjun Li, Fuhua Yu, Zefen Wang, Xu Gao, Zhiqiang Li

Supplementary Methods

Patients Enrollment Process for Constructing Prediction Model.

To develop a prediction model for IDH mutation status, patients were accrued at Zhongnan Hospital (ZNH), General Hospital of Northern Theater Command (NTCGH), Liaocheng Peoples Hospital (LPH), and Chinese PLA General Hospital (PLAGH). 435 patients who underwent preoperative MRI for newly diagnosed gliomas from June 2017 to June 2021 at ZNH were considered for inclusion. The inclusion criteria were as follows: (i) pathologically confirmed glioma, (ii) known IDH mutation status, (iii) preoperative MRI inclusive of postcontrast T1-weighted (T1wC), T1-weighted (T1w), T2-weighted (T2w), and fluid-attenuated inversion recovery (FLAIR) images, and (iv) age ≥18 years. The exclusion criteria were as follows: (i) history of biopsy or surgery for brain tumor (n=78), (ii) the absence of T1wC, T1w, T2w, or FLAIR images (n=64), and/or (iii) unknown IDH status (n=32). 261 patients at ZNH were definitively enrolled (ZNH set). A total of 121 patients from June 2017 to June 2021 at NTCGH, 104 patients from June 2017 to June 2021 at LPH, 30 patients from January 2022 to August 2022 at PLAGH, and 148 patients from The Cancer Imaging Archive (TCIA set) were enrolled in accordance with the same criteria.

The patients from ZNH, NTCGH, and LPH were used for model development (n=414) and internal test (n=72) sets, and the development set was subsequently divided into the training (n=340) and validation (n=74) sets. PLAGH (n=30) set was used as external test set.

Evaluation for Isocitrate Dehydrogenase (IDH) Mutation Status

Isocitrate dehydrogenase (IDH) R132H mutation was detected through immunohistochemistry (IHC) analysis in four in-house sets: ZNH, NTCGH, LPH, and PLAGH sets. To validate or exclude negative IDH-mutation results obtained through IHC in lower-grade gliomas or patients diagnosed at age 55 or younger, sequencing was performed. According to the guidelines of the European Association for Neuro-Oncology [1], patients with a GBM pathological diagnosis and negative IDH-mutation based on IHC who were over the age of 55 were classified as IDH-wildtype. In the case of the TCGA cohort, histological findings and tissue grading submitted to TCGA underwent confirmation by neuropathology review [2].

MRI Image Processing

T1wC images were resampled to a 1mm isovoxel using the 'interpolation' function from the 'SciPy' python package. The resampled T1w, T2w, and FLAIR images were then registered to the isovoxel T1wC using the 'FLIRT' tool from FSL (https://fsl.fmrib.ox.ac.uk/fsl/fslwiki) with 12 degrees of freedom and normalized mutual information as the cost function. The registered and skull-stripped T1wC, T1w, T2w, and FLAIR images were corrected for bias using the ANTs package [3] with the N4 bias correction method. Subsequently, image signal intensity normalization was performed by subtracting the mean value and dividing by the standard deviation of the signal intensity from the entire brain tissue. Finally, the images were resampled to a size of 256 × 256.

The ground truth whole tumor segmentation was performed by a neuroradiologist (Mingjun Li) with 8 years of experience and confirmed by another neurosurgeon (Yilei Xiao) with 11 years of experience. The segmentation of the ground truth was performed using semi-automatic methods based on distinct tumor signal intensity with the open-source software 3D Slicer (https://www.slicer.org/).

Development and Structure of Prediction Model

Vision Transformer

Vision Transformer (ViT) was utilized as the backbone architecture for both pre-training and the classification task. A ViT consists of three main components: the patch embedding layer, position embedding, and Transformer blocks [4].

(i) Patch Embedding: The patch embedding layer needs to transform high-dimensional data into sequences. For a 3D volume $X \in R^{H \times W \times D \times C}$, it is reshaped into a sequence of flattened 3D patches $X_p \in R^{N \times (P^{\wedge}3 \cdot C)}$ (C is the input channel, (H, W, D) is the input resolution, (P, P, P) is the patch resolution, and $N = HWD/P^3$ is the number of patches). A trainable linear projection is then applied to map them to patch embeddings.

(ii) Position Embedding: In order to retain positional information, position embeddings are added to the patch embeddings. The standard ViT utilizes 1D learnable position embeddings.

(iii) Transformer Block: The Transformer block consists of alternating layers of multiheaded self-attention [5] and MLP blocks.

Self-Pre-training with Masked Autoencoders

The encoder, decoder and loss function in Masked Autoencoders (MAE) [6] were illustrated as follows:

(i) Encoder in MAE. The input image is first divided into non-overlapping patches. These patches are then randomly grouped into visible patches and masked patches. The encoder in MAE, which is a ViT, only operates on the visible patches.

(ii) Decoder in MAE. The decoder in MAE takes the full set of tokens as input, including patch-wise representations from the encoder and learnable mask tokens placed in the positions of masked patches. By adding positional embeddings to all the input tokens, the decoder is able to restore the patches in each specific masked position. To reduce the pre-training budget, the decoder is designed to be more lightweight.

(iii) Loss function in MAE. MAE is trained using a reconstruction loss by predicting the pixel values of the masked patches.

(iv) MAE was pre-trained with a batch size of 32, weight decay of 0.05, and training epoch of 800 using Stochastic Gradient Descent (SGD) for optimization. Intermediate weights were saved for each 100 epochs and tested to determine the one with the best reconstruction performance.

Architecture for classification task

A linear classifier was added after extracting the class token output from the Vision Transformer (ViT). To address the classification task, we utilized T1wC, T1w, T2w, and FLAIR images as inputs to the network. The patch size was set to 16 × 16. Each image type was individually processed through the network, generating corresponding feature maps. Averaging the four feature maps, we obtained a combined map before performing the final classification. To ensure stable training of the classification head, we implemented a layer-wise learning rate decay strategy. During training, we used a batch size of 16 and employed stochastic gradient descent (SGD) for optimization. The maximum number of training epochs was set to 150, and we saved the model with the highest accuracy on the validation set.

Fabrication of CRISPR/Cas12a-based AIGS

A compact detection device, based on CRISPR/Cas12a and designed using SolidWorks and Mentor software, was created. The device measures 160×210×155 mm and incorporates three main functions: temperature control, photoexcitation, and fluorescence signal recording. To achieve these functions, the device contains five modules: (i) A temperature control system. (ii) Optical components, such as a 470-nm LED and 470- and 520-nm band-pass filters. The 470-nm LED is used for fluorescence excitation, while the 470-nm band-pass filter filters stray light and the 520-nm band-pass filter reduces background interference. (iii) A photodetector and signal processing board for fluorescence collection. (iv) An MCU and various auxiliary components for generating curves based on collected fluorescence. (v) A control board for temperature regulation and LED activation.

To enable intelligent operation, an app was developed using Android Studio. The app serves two main functions: (i) Fluorescence signal analysis. This involves recording and displaying the fluorescence intensities for each channel through the serial port. (ii) Timing control. Serial port commands are used to control the timing sequence of each component. The workflow of the app follows this sequence: send a command to the control board > initiate rapid PCR > 30 minutes later, open the valve of the reaction tube and shake it > simultaneously record the fluorescence signals > 30 minutes later, switch off the heat-controlled system and LED light.

The 470-nm LED, 470- and 520-nm band-pass filters were purchased from CREE, while the photodetector was purchased from OSRAM. The shell of the device was manufactured by Suzhou Daqian Model, and the control motherboard was produced by Jiangsu Jialichuang Electronic Technology.

The computation procedure of GradCAM importance

In the heatmaps generated using GradCAM, the intensity of the color was used to quantify the important region. The scaled intensity was calculated by subtracting the Green value from the Red value. It was normalized to a range of 0–1. In addition, the intensity of some areas not covered by GradCAM was considered −1. The formulas are as follows:

$X_i = (\text{Red value})_i - (\text{Green value})_i$,

$(\text{Grad-CAM importance})_i = (X_i - X_{min})/(X_{max} - X_{min})$.

Then, the correlation between GradCAM importance and tumor cell percentage was analyzed using Spearman's rank correlation coefficient.

Droplet Digital PCR

In order to compare the molecular characteristics of different samples from patient 06, droplet digital PCR (ddPCR) was conducted using the protocol provided by the manufacturer, Sniper Medical Technology Co., Ltd. A total of 80ng DNA was used as input for the experiment. The ddPCR reactions were carried out in 22 μL volumes, with 500 nM of primers and 250 nM of both a fluorescein (FAM)–labeled R132 wildtype probe and a victoria (VIC)–labeled mutant R132H probe. The reaction mixture was then partitioned into nanoliter-sized droplets using the automated DQ24 Droplet Generator. PCR amplification was performed with the following cycling conditions: an initial stage of enzyme activation at 95ºC for 15 minutes, followed by 40 cycles of denaturation at 95ºC for 15 seconds and annealing/extension at 60ºC for 1 minute. The reaction was completed with a final stage of enzyme deactivation at 98ºC for 10 minutes. To identify droplets positive for FAM and/or VIC signal, the PCR product was analyzed using a droplet reader. Poisson distribution analysis was used to quantify the fractional abundance of the IDH1-R132H mutation in the sample:

The primer sequences used for ddPCR are listed below.

ddPCR-IDH1-F	5'-CGGTCTTCAGAGAAGCCATT-3'
ddPCR-IDH1-R	5'-ATTCTTATCTTTTGGTATCTACACC-3'
FAM-WT	5'-ATCATAGGTCGTCATGCTTAT-3'
VIC-R132H	5'-ATCATAGGTCATCATGCTTAT-3'

Whole Exome Sequencing

To investigate the heterogeneity in IDH-wildtype gliomas, we performed whole exome sequencing (WES) on the tumor samples. The tumor samples were processed at Harbin Xingyun Biological Information Technology Development Co., Ltd., where genomic DNA from the tumor was fragmented and underwent end-repair, a-tailing, adapter ligation, and PCR. Paired-end (PE) sequencing was then conducted using the DNBSEQ-T7RS instrument, generating 150 bases from each end of the fragments. The resulting PE reads were aligned to the human genome (hg38) using the Burrows-Wheeler Aligner, and the sam files were converted to bam format and sorted by coordinates using Samtools. Duplicate reads were marked using Samblaster, and base quality score recalibration was performed with GAKT. The quality of the alignment was evaluated using Qualimap. To identify somatic variants, including point mutations, small insertions, and deletions, Mutect2 in GATK, CNVkit, and Manta were used. The resulting VCF files were annotated with VEP to extract variant-related information, such as the start and end position of the gene. Furthermore, the purity of each tumor sample was estimated based on the CNV data using the R package ABSOLUTE (v.1.0.6) [7].

To identify candidate somatic mutations, an alignment filter was applied to exclude quality failed reads, unpaired reads, and poorly mapped reads in the tumor. A base quality filter was applied to include bases with a reported Phred quality score >20 for the tumor samples. Somatic mutation candidates were identified based on the following filter conditions: (i) mutations with a low depth (allele depth, <20) were excluded; (ii) mutations with a low allele fraction of alternate alleles in the tumor (allele frequency, <0.1) were excluded; (iii) mutations with supporting reads greater than or equal to 5 were selected; (iv) mutations with a PASS in the filter were selected; (v) mutations with a minor allele frequency (MAF) less than 0.0001 in populations were excluded; (vi) mutations reported as benign or likely benign were excluded; (vii) intronic and silent changes were excluded, while mutations resulting in missense mutations, nonsense mutations, frameshifts, or splice site alterations were retained. The qualified mutations from each sample were used to construct phylogenetic trees. Specifically, sequences encompassing the mutations in a total length of 20 bp were extracted to infer the phylogeny among samples of each patient, using the maximum parsimony algorithm in MEGA [8]

Supplementary Tables

Supplementary Table 1. IDH status of the patients for constructing predictive model.

IDH status	ZNH	NTCGH	LPH	PLAGH	TCIA set
Wildtype	142	46	68	20	91
IDH1-R132H	115	74	38	10	51
IDH1- R132S	1	0	0	0	3
IDH1-R132G	0	0	0	0	1
IDH1-R132C	0	0	0	0	1
IDH2-R172K	3	1	0	0	0
IDH2-R172S	0	0	0	0	1
Total	261	121	104	30	148

Abbreviations: IDH, isocitrate dehydrogenase; ZNH, Zhongnan Hospital; NTCGH, General Hospital of Northern Theater Command; LPH, Liaocheng Peoples Hospital; PLAGH, Chinese PLA General Hospital; TCIA, The Cancer Imaging Archive.

Supplementary Table 2. Clinical characteristics of the patients for constructing prediction model.

Characteristic	Development set (n=413)	Internal test set (n=73)	PLAGH set (n=30)	TCIA set (n=148)	P-value
Age (y), Mean ± SD	52.51±12.85	49.66±11.79	50.10±10.28	51.46±15.62	0.30 [a]
Sex, no. (%)					0.56 [b]
Female	172 (41.65)	33 (45.21)	13 (43.33)	71 (47.97)	
Male	241 (58.35)	40 (54.79)	17 (56.67)	76 (51.35)	
IDH status, no. (%)					0.12 [b]
Mutated	199 (48.18)	32 (43.84)	10 (33.33)	57 (38.51)	
Wildtype	214 (51.82)	41 (56.16)	20 (66.67)	91 (61.49)	
1p/19q co-deletion status, no. (%)					**0.02** [b]
Co-deleted	83 (20.10)	11 (15.07)	6 (20.00)	13 (8.78)	
Intact	330 (79.90)	62 (84.93)	24 (80.00)	130 (87.84)	
2016 WHO Grade, no. (%)					0.49 [b]
II	114 (27.60)	18 (24.66)	12 (40.00)	54 (36.49)	
III	106 (25.67)	19 (26.03)	4 (13.33)	37 (25.00)	
IV	193 (46.73)	36 (49.32)	20 (66.67)	83 (56.08)	
Extent of resection, no. (%)					0.50 [b]
GTR	204 (49.39)	35 (47.95)	18 (60.00)	-	
Non-GTR	209 (50.61)	38 (52.05)	12 (40.00)	-	
Recurrence time, no. (%)					0.99 [b]
< 1 year	202 (48.91)	34 (46.58)	15 (50.00)	-	

⩾1 year	110 (26.63)	18 (24.66)	8 (26.67)	-
Unknown	101 (24.46)	21 (28.77)	7 (23.33)	-

a, ANOVA test; b, Chi-square test.

Abbreviations: IDH, isocitrate dehydrogenase; WHO, World Health Organization; GTR, gross total resection.

Supplementary Table 3. Clinical characteristics of the patients for evaluating the performance of CRISPR/Cas12a-based AIGS.

Characteristic	NTCGH cohort (n=20)	LPH cohort (n=91)
Sample type	Frozen tissue	FFPE tissue
Age (y), Mean ± SD	56.00±7.85	54.16±12.21
Sex, no. (%)		
Female	8 (40.00)	44 (48.35)
Male	12 (60.00)	47 (51.65)
IDH status, no. (%)		
Mutated	12 (60.00)	50 (54.95)
Wildtype	8 (40.00)	41 (45.05)
Histology, no. (%)		
Glioblastoma	8 (40.00)	62 (68.13)
Astrocytoma	7 (35.00)	21 (23.08)
Oligodendroglioma	5 (25.00)	8 (8.79)
2021 WHO Grade		
2	5 (25.00)	80 (87.91)
3	5 (25.00)	6 (6.59)
4	10 (50.00)	5 (5.49)

Abbreviations: CRISPR, clustered regularly interspaced short palindromic repeats; AIGS, automatic integrated gene detection system; FFPE, formalin-fixed and parrffin-embedded; IDH, isocitrate dehydrogenase.

Supplementary Table 4. The characteristics of the patients with multiple biopsies.

Patient	Age, y	Sex	Histology	2021WHO Grade	IDH Status	Lesion Site	Contrast Enhancement
01	57	Female	Astrocytoma	4	Mutant	Right temporal	Yes
02	71	Female	Glioblastoma	4	Wildtype	Left frontoparietal	Yes
03	59	Male	Glioblastoma	4	Wildtype	Right frontotemporal	Yes
04	28	Female	Astrocytoma	3	Mutant	Right frontal	No
05	31	Female	Astrocytoma	2	Mutant	Left frontal	No
06	31	Male	Oligodendroglioma	3	Mutant	Right frontotemporal	Yes
07	69	Female	Glioblastoma	4	Wildtype	Right parietal	Yes
08	32	Female	Oligodendroglioma	2	Mutant	Left frontotemporal	No
09	47	Male	Astrocytoma	3	Mutant	Right temporal	No
10	29	Female	Oligodendroglioma	2	Mutant	Left frontal	No
11	68	Male	Astrocytoma	2	Mutant	Right parietal	No
12	49	Female	Glioblastoma	4	Wildtype	Right parietal-temporal	No
13	56	Male	Glioblastoma	4	Wildtype	Right parietal-temporal	Yes

Abbreviations: WHO, World Health Organization; IDH, isocitrate dehydrogenase.

Supplementary Table 5. Ablation Study for MAE pretraining on mask ratios and epochs.

Mask ratio	Pre-train Epochs	AUC for internal test set
87.5%	100	0.8412
	200	0.8635
	500	0.8804
	1000	0.8917
75%	100	0.8932
	200	0.9144
	500	0.9301
	1000	0.9324
50%	100	0.8671
	200	0.8829
	500	0.9001
	1000	0.9010

AUC, area under the receiver operating characteristic curve.

Supplementary Table 6. The prediction status for IDH1/2 variants in MRI model.

Variant	Wild type (prediction)	Mutant (prediction)	Accuracy
IDH1-R132H			
Internal test set (n = 31)	3	28	0.90
PLAGH set (n = 10)	1	9	0.90
TCIA set (n = 51)	14	40	0.78
IDH1-R132S			
Internal test set (n = 0)	-	-	-
PLAGH set (n = 0)	-	-	-
TCIA set (n = 3)	1	2	0.67
IDH1-R132G			
Internal test set (n = 0)	-	-	-
PLAGH set (n = 0)	-	-	-
TCIA set (n = 1)	0	1	1.00
IDH1-R132C			
Internal test set (n = 0)	-	-	-
PLAGH set (n = 0)	-	-	-
TCIA set (n = 1)	0	1	1.00
IDH2-R172K			
Internal test set (n = 1)	0	1	1.00
PLAGH set (n = 0)	-	-	-
TCIA set (n = 0)	-	-	-
IDH2-R172S			
Internal test set (n = 0)	-	-	-
PLAGH set (n = 0)	-	-	-
TCIA set (n = 1)	1	0	0.00

Abbreviations: IDH, isocitrate dehydrogenase; ZNH, Zhongnan Hospital; NTCGH, General Hospital of Northern Theater Command; LPH, Liaocheng Peoples Hospital; PLAGH, Chinese PLA General Hospital; TCIA, The Cancer Imaging Archive.

Supplementary Table 7. A cohort of FFPE cases (n=91) with known mutation status was assayed with CRISPR/Cas12a-based AIGS.

Patient	Sex	Age, y	Histology	2021 WHO Grade	Molecular biomarkers	IDH1-R132H IHC	NGS	CRISPR-based AIGS
LPH-P01	Male	61	Glioblastoma	4	IDH wildtype	Negative	IDH wildtype	IDH wildtype
LPH-P02	Female	66	Glioblastoma	4	IDH wildtype	Negative	IDH wildtype	IDH wildtype
LPH-P03	Female	67	Glioblastoma	4	IDH wildtype	Negative	IDH wildtype	IDH wildtype
LPH-P04	Female	46	Glioblastoma	4	IDH wildtype	Negative	IDH wildtype	IDH wildtype
LPH-P05	Female	46	Oligodendroglioma	3	IDH1 mutant, 1p/19q co-deleted	Negative	IDH1-R132H	IDH1-R132H
LPH-P06	Female	51	Astrocytoma	4	IDH1 mutant, 1p/19q intact	Positive	IDH1-R132H	IDH1-R132H
LPH-P07	Female	76	Astrocytoma	4	IDH1 mutant, 1p/19q intact	Negative	IDH1-R132H	IDH1-R132H
LPH-P08	Female	37	Glioblastoma	4	IDH wildtype	Negative	IDH wildtype	IDH wildtype
LPH-P09	Male	52	Glioblastoma	4	IDH wildtype	Negative	IDH wildtype	IDH wildtype
LPH-P10	Female	63	Glioblastoma	4	IDH wildtype	Negative	IDH wildtype	IDH wildtype
LPH-P11	Female	50	Glioblastoma	4	IDH wildtype	Positive	IDH wildtype	IDH wildtype
LPH-P12	Male	34	Glioblastoma	4	IDH wildtype	Negative	IDH wildtype	IDH wildtype

LPH-P13	Male	66	Astrocytoma	3	IDH1 mutant, 1p/19q intact	Positive	IDH1-R132H	IDH1-R132H
LPH-P14	Male	44	Glioblastoma	4	IDH wildtype	Negative	IDH wildtype	IDH wildtype
LPH-P15	Male	64	Oligodendroglioma	3	IDH1 mutant, 1p/19q co-deleted	Positive	IDH1-R132H	IDH1-R132H
LPH-P16	Male	51	Oligodendroglioma	3	IDH1 mutant, 1p/19q co-deleted	Positive	IDH1-R132H	IDH1-R132H
LPH-P17	Female	68	Glioblastoma	4	IDH wildtype	Negative	IDH wildtype	IDH wildtype
LPH-P18	Female	56	Glioblastoma	4	IDH wildtype	Negative	IDH wildtype	IDH wildtype
LPH-P19	Female	43	Glioblastoma	4	IDH wildtype	Negative	IDH wildtype	IDH wildtype
LPH-P20	Male	48	Oligodendroglioma	2	IDH1 mutant, 1p/19q co-deleted	Positive	IDH1-R132H	IDH1-R132H
LPH-P21	Female	47	Glioblastoma	4	IDH wildtype	Negative	IDH wildtype	IDH wildtype
LPH-P22	Male	62	Glioblastoma	4	IDH wildtype	Negative	IDH wildtype	IDH wildtype
LPH-P23	Male	43	Glioblastoma	4	IDH wildtype	Negative	IDH wildtype	IDH wildtype
LPH-P24	Female	52	Glioblastoma	4	IDH wildtype	Negative	IDH wildtype	IDH wildtype
LPH-P25	Male	68	Glioblastoma	4	IDH wildtype	Negative	IDH wildtype	IDH wildtype
LPH-P26	Male	45	Glioblastoma	4	IDH wildtype	Negative	IDH wildtype	IDH wildtype
LPH-P27	Male	39	Glioblastoma	4	IDH wildtype	Positive	IDH wildtype	IDH wildtype
LPH-P28	Female	37	Glioblastoma	4	IDH wildtype	Negative	IDH wildtype	IDH wildtype
LPH-P29	Male	66	Glioblastoma	4	IDH wildtype	Negative	IDH wildtype	IDH wildtype

LPH-P30	Male	17	Glioblastoma	4	IDH wildtype	Negative	IDH wildtype	IDH wildtype
LPH-P31	Male	53	Glioblastoma	4	IDH wildtype	Negative	IDH wildtype	IDH wildtype
LPH-P32	Male	62	Glioblastoma	4	IDH wildtype	Negative	IDH wildtype	IDH wildtype
LPH-P33	Male	37	Astrocytoma	4	IDH1 mutant, 1p/19q intact	Positive	IDH1-R132H	IDH1-R132H
LPH-P34	Female	71	Glioblastoma	4	IDH wildtype	Negative	IDH wildtype	IDH wildtype
LPH-P35	Male	70	Astrocytoma	4	IDH1 mutant, 1p/19q intact	Positive	IDH1-R132H	IDH1-R132H
LPH-P36	Male	40	Astrocytoma	2	IDH1 mutant, 1p/19q intact	Positive	IDH1-R132H	IDH1-R132H
LPH-P37	Female	60	Glioblastoma	4	IDH wildtype	Negative	IDH wildtype	IDH wildtype
LPH-P38	Female	62	Glioblastoma	4	IDH wildtype	Negative	IDH wildtype	IDH wildtype
LPH-P39	Female	41	Glioblastoma	4	IDH wildtype	Negative	IDH wildtype	IDH wildtype
LPH-P40	Male	48	Astrocytoma	4	IDH1 mutant, 1p/19q intact	Negative	IDH1-R132H	IDH1-R132H
LPH-P41	Male	70	Astrocytoma	4	IDH1 mutant, 1p/19q intact	Negative	IDH1-R132H	IDH1-R132H
LPH-P42	Male	53	Glioblastoma	4	IDH wildtype	Negative	IDH wildtype	IDH wildtype
LPH-P43	Female	59	Glioblastoma	4	IDH wildtype	Negative	IDH wildtype	IDH wildtype
LPH-P44	Female	63	Glioblastoma	4	IDH wildtype	Negative	IDH wildtype	IDH wildtype
LPH-P45	Female	66	Glioblastoma	4	IDH wildtype	Negative	IDH wildtype	IDH wildtype
LPH-P46	Male	67	Glioblastoma	4	IDH wildtype	Negative	IDH wildtype	IDH wildtype

LPH-P47	Male	59	Astrocytoma	4	IDH2 mutant, 1p/19q intact	Negative	IDH2-R172K	IDH2-R172K
LPH-P48	Female	54	Glioblastoma	4	IDH wildtype	Negative	IDH wildtype	IDH wildtype
LPH-P49	Female	62	Astrocytoma	4	IDH1 mutant, 1p/19q intact	Positive	IDH1-R132H	IDH1-R132H
LPH-P50	Male	62	Astrocytoma	4	IDH1 mutant, 1p/19q intact	Negative	IDH1-R132H	IDH1-R132H
LPH-P51	Male	66	Astrocytoma	4	IDH1 mutant, 1p/19q intact	Negative	IDH1-R132H	IDH1-R132H
LPH-P52	Female	66	Glioblastoma	4	IDH wildtype	Negative	IDH wildtype	IDH wildtype
LPH-P53	Male	63	Glioblastoma	4	IDH wildtype	Negative	IDH wildtype	IDH wildtype
LPH-P54	Male	73	Astrocytoma	4	IDH2 mutant, 1p/19q intact	Negative	IDH2-R172K	IDH2-R172K
LPH-P55	Female	45	Glioblastoma	4	IDH wildtype	Negative	IDH wildtype	IDH wildtype
LPH-P56	Female	68	Glioblastoma	4	IDH wildtype	Negative	IDH wildtype	IDH wildtype
LPH-P57	Male	50	Oligodendroglioma	2	IDH1 mutant, 1p/19q co-deleted	Positive	IDH1-R132H	IDH1-R132H
LPH-P58	Female	29	Oligodendroglioma	3	IDH1 mutant, 1p/19q co-deleted	Positive	IDH1-R132H	IDH1-R132H
LPH-P59	Male	33	Glioblastoma	4	IDH wildtype	Negative	IDH wildtype	IDH wildtype
LPH-P60	Male	51	Glioblastoma	4	IDH wildtype	Negative	IDH wildtype	IDH wildtype
LPH-P61	Female	62	Glioblastoma	4	IDH wildtype	Negative	IDH wildtype	IDH wildtype
LPH-P62	Female	37	Glioblastoma	4	IDH wildtype	Negative	IDH wildtype	IDH wildtype
LPH-P63	Female	49	Astrocytoma	4	IDH1 mutant, 1p/19q intact	Positive	IDH1-R132H	IDH1-R132H

LPH-P64	Female	49	Astrocytoma	4	IDH1 mutant, 1p/19q intact	Positive	IDH1-R132H	IDH1-R132H
LPH-P65	Male	64	Glioblastoma	4	IDH wildtype	Negative	IDH wildtype	IDH wildtype
LPH-P66	Female	55	Oligodendroglioma	2	IDH1 mutant, 1p/19q co-deleted	Positive	IDH1-R132H	IDH1-R132H
LPH-P67	Male	59	Glioblastoma	4	IDH wildtype	Negative	IDH wildtype	IDH wildtype
LPH-P68	Female	45	Astrocytoma	4	IDH1 mutant, 1p/19q intact	Positive	IDH1-R132H	IDH1-R132H
LPH-P69	Female	70	Glioblastoma	4	IDH wildtype	Negative	IDH wildtype	IDH wildtype
LPH-P70	Male	54	Glioblastoma	4	IDH wildtype	Negative	IDH wildtype	IDH wildtype
LPH-P71	Male	54	Oligodendroglioma	2	IDH1 mutant, 1p/19q co-deleted	Positive	IDH1-R132H	IDH1-R132H
LPH-P72	Male	74	Glioblastoma	4	IDH wildtype	Negative	IDH wildtype	IDH wildtype
LPH-P73	Female	50	Glioblastoma	4	IDH wildtype	Negative	IDH wildtype	IDH wildtype
LPH-P74	Male	52	Glioblastoma	4	IDH wildtype	Negative	IDH wildtype	IDH wildtype
LPH-P75	Female	49	Astrocytoma	4	IDH1 mutant, 1p/19q intact	Negative	IDH1-R132H	IDH1-R132H
LPH-P76	Female	39	Glioblastoma	4	IDH wildtype	Negative	IDH wildtype	IDH wildtype
LPH-P77	Male	41	Astrocytoma	4	IDH1 mutant, 1p/19q intact	Positive	IDH1-R132H	IDH1-R132H
LPH-P78	Female	64	Glioblastoma	4	IDH wildtype	Negative	IDH wildtype	IDH wildtype
LPH-P79	Male	36	Astrocytoma	4	IDH1 mutant, 1p/19q intact	Positive	IDH1-R132H	IDH1-R132H
LPH-P80	Male	63	Glioblastoma	4	IDH wildtype	Negative	IDH wildtype	IDH wildtype

LPH-P81	Male	55	Glioblastoma	4	IDH wildtype	Negative	IDH wildtype	IDH wildtype
LPH-P82	Female	44	Glioblastoma	4	IDH wildtype	Negative	IDH wildtype	IDH wildtype
LPH-P83	Male	44	Glioblastoma	4	IDH wildtype	Negative	IDH wildtype	IDH wildtype
LPH-P84	Female	61	Glioblastoma	4	IDH wildtype	Negative	IDH wildtype	IDH wildtype
LPH-P85	Male	68	Glioblastoma	4	IDH wildtype	Negative	IDH wildtype	IDH wildtype
LPH-P86	Male	71	Glioblastoma	4	IDH wildtype	Negative	IDH wildtype	IDH wildtype
LPH-P87	Female	47	Glioblastoma	4	IDH wildtype	Negative	IDH wildtype	IDH wildtype
LPH-P88	Female	71	Glioblastoma	4	IDH wildtype	Negative	IDH wildtype	IDH wildtype
LPH-P89	Female	40	Astrocytoma	3	IDH1 mutant, 1p/19q intact	Positive	IDH1-R132H	IDH1-R132H
LPH-P90	Male	29	Astrocytoma	4	IDH1 mutant, 1p/19q intact	Positive	IDH1-R132H	IDH1-R132H
LPH-P91	Male	65	Glioblastoma	4	IDH wildtype	Negative	IDH wildtype	IDH wildtype

Abbreviations: FFPE, formalin-Fixed and Paraffin-Embedded; WHO, World Health Organization; IDH, isocitrate dehydrogenase; IHC, immunohistochemistry; CRISPR, clustered regularly interspaced short palindromic repeats; AIGS, automatic integrated gene detection system.

Supplementary Figures

Supplementary Fig. S1. Flow diagram of the patient enrollment for constructing the predictive model.

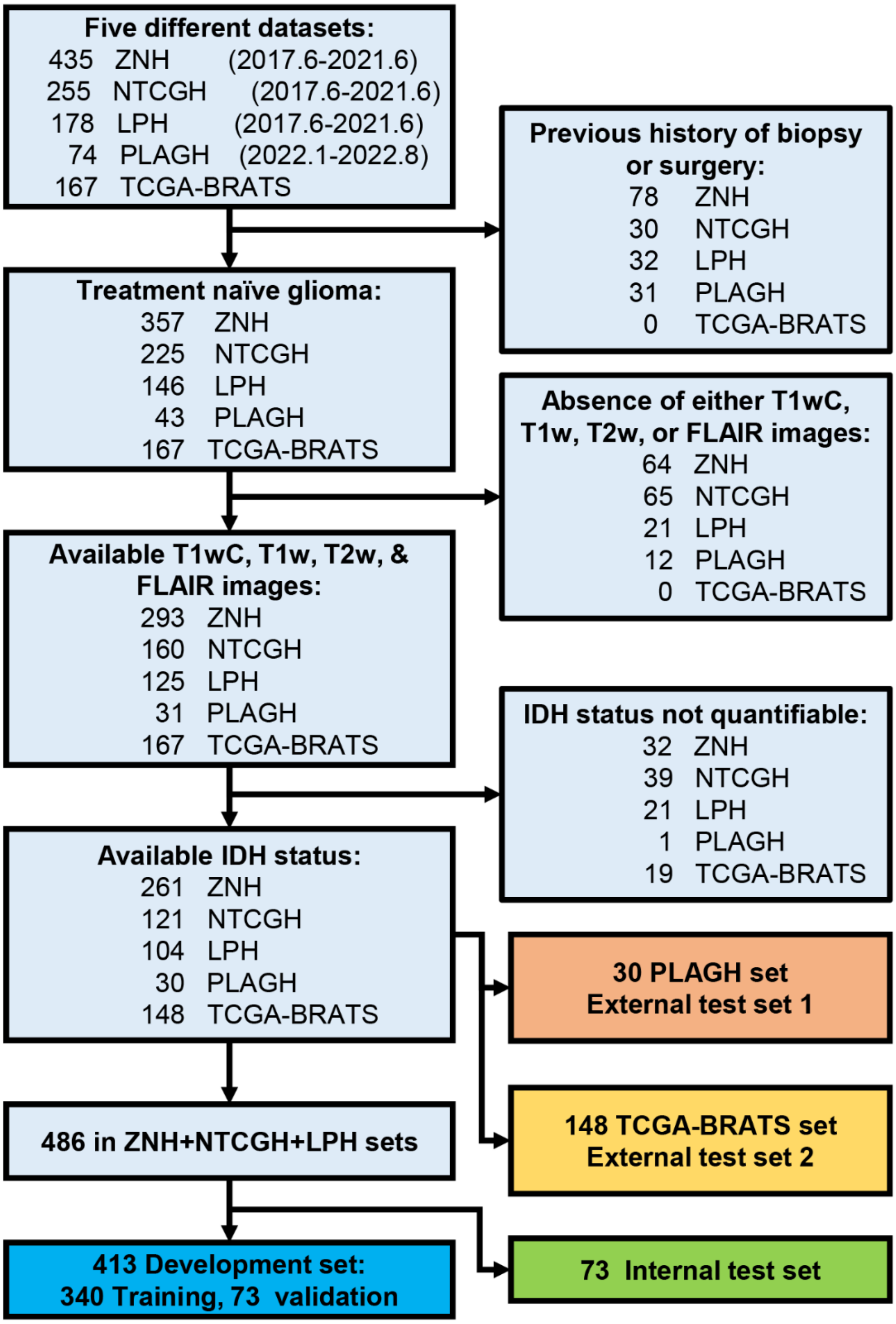

Supplementary Fig. S2. Reconstructions of MRI images by MAE pre-trained. *First row:* the original images; *second row*: the masked images by black patches with masking ratio of 75%; *third row:* the reconstruction results of MAE.

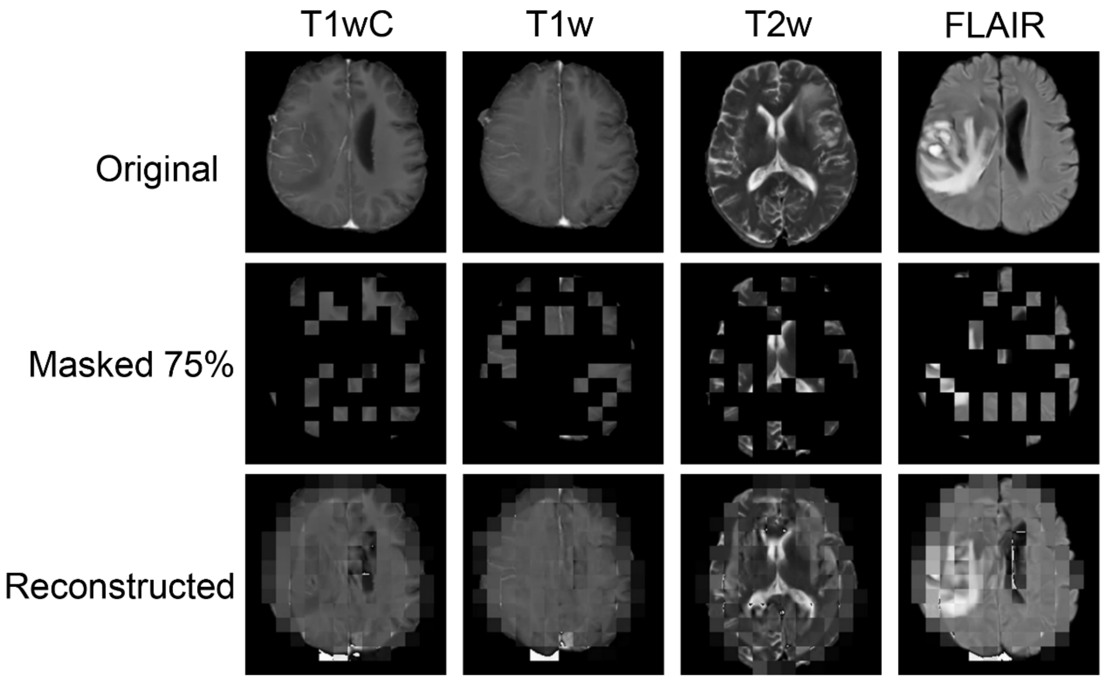

Abbreviations: T1wC, contrast-enhanced T1 weighted imaging; T1w, T1 weighted imaging; T2w, T2 weighted imaging; FLAIR, fluid-attenuated inversion recovery.

Supplementary Fig. S3. GradCAM analysis of the right and wrong predicted patients with the model. Analysis of GradCAM maps for patients with incorrect predictions revealed that the model's focus extended beyond the tumor region, in contrast to accurately predicted cases where the model predominantly concentrated on the lesion.

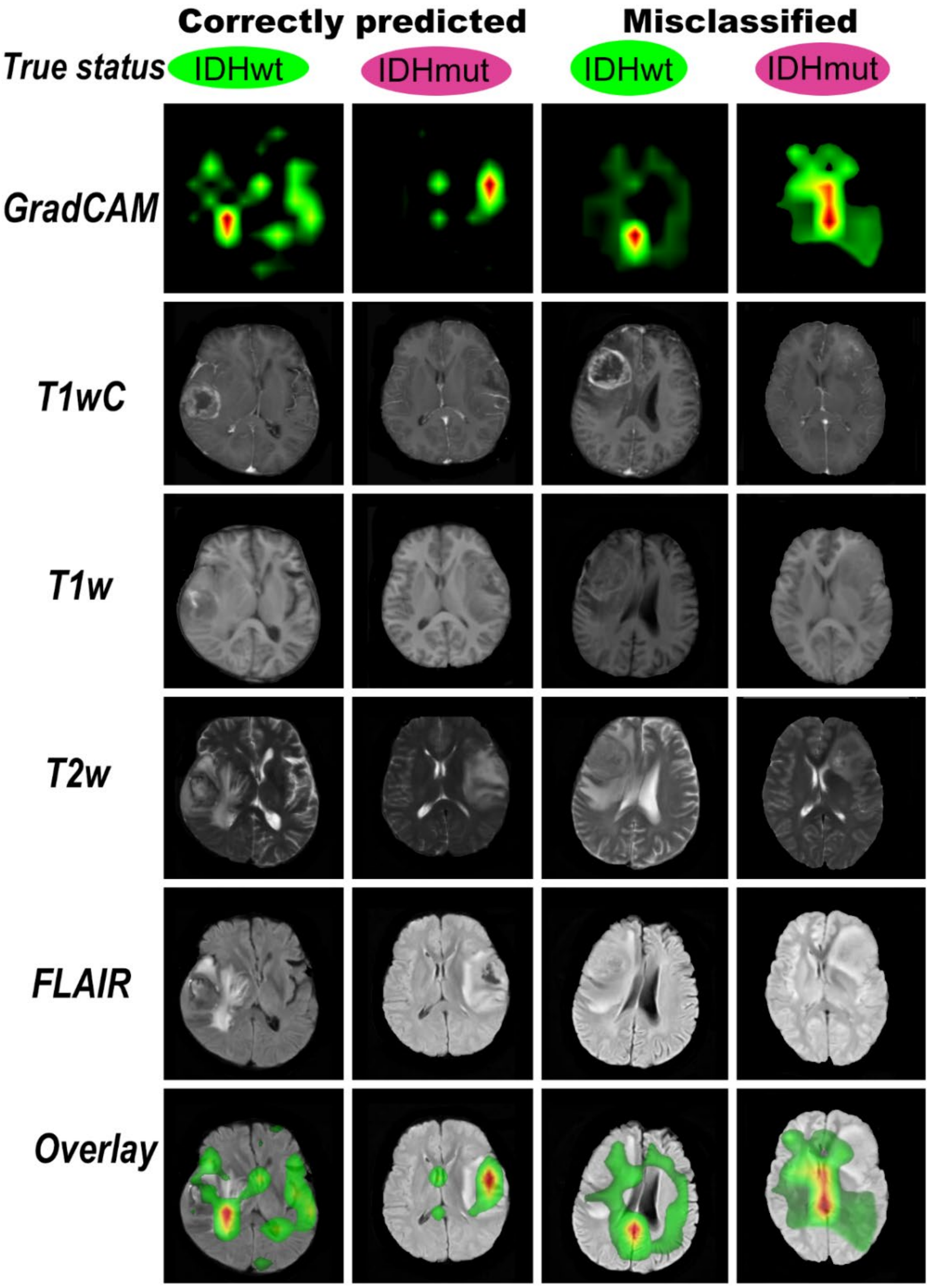

Abbreviations: GradCAM, Gradient-weighted Class Activation Mapping; T1wC, contrast-enhanced T1 weighted imaging; T1w, T1 weighted imaging; T2w, T2 weighted imaging;

FLAIR, fluid-attenuated inversion recovery.

Supplementary Fig. S4. Primers selections for rapid PCR. The sequences of upstream and downstream primers for (A) IDH1 and (C) IDH2. Gel images of rapid PCR amplification for (B) IDH1 and (D) IDH2 with 40ng DNA sample as input.

A

IDH1-F1	5'-CTGCAGAAGCTATAAAGAAGCATAATGT-3'
IDH1-R1	5'-CAAAATCACATTATTGCCAACATGACTTAC-3'
IDH1-F2	5'-GCAGAAGCTATAAAGAAGCATAATGTTG-3'
IDH1-R2	5'-AAATCACATTATTGCCAACATGACTTAC-3'
IDH1-F3	5'-ATGATTTAGGCATAGAGAATCGTGATGA-3'
IDH1-R3	5'-AATTTCATACCTTGCTTAATGGGTGTAGTA-3'
IDH1-F4	5'-TCACCAAATGGCACCATACGAAATA-3'
IDH1-R4	5'-CATACCTTGCTTAATGGGTGTAGTA-3'

B

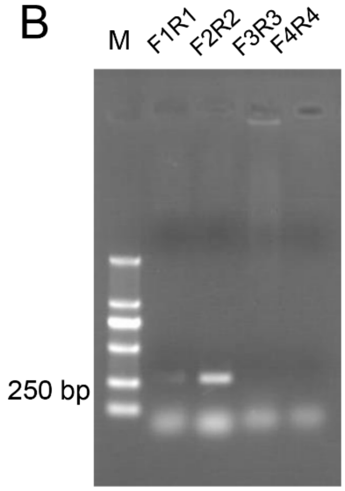

C

IDH2-F1	5'-CCCGGTCTGCCACAAAGTC-3'
IDH2-R1	5'-GGACCACTATTATCTCTGTCCTCA-3'
IDH2-F2	5'-ACCATTTTGAAAGTGCCGGC-3'
IDH2-R2	5'-GTCCTCACAGAGTTCAAGCTGA-3'
IDH2-F3	5'-CGGTCTGCCACAAAGTCTGT-3'
IDH2-R3	5'-GTGGGACCACTATTATCTCTGTCC-3'
IDH2-F4	5'-CATCTTTTGGGGTGAAGACCA-3'
IDH2-R4	5'-CCTCACAGAGTTCAAGCTGAA-3'

D

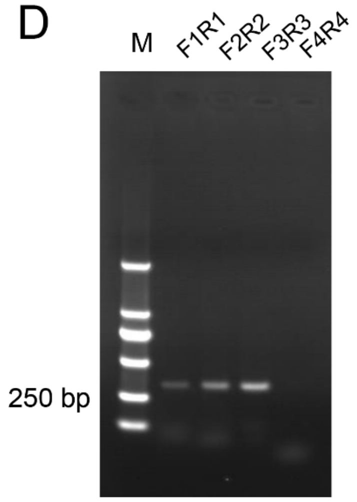

Abbreviations: IDH, isocitrate dehydrogenase.

Supplementary Fig. S5. Novel CRISPR/Cas12a-based AIGS with eight detecting channels.

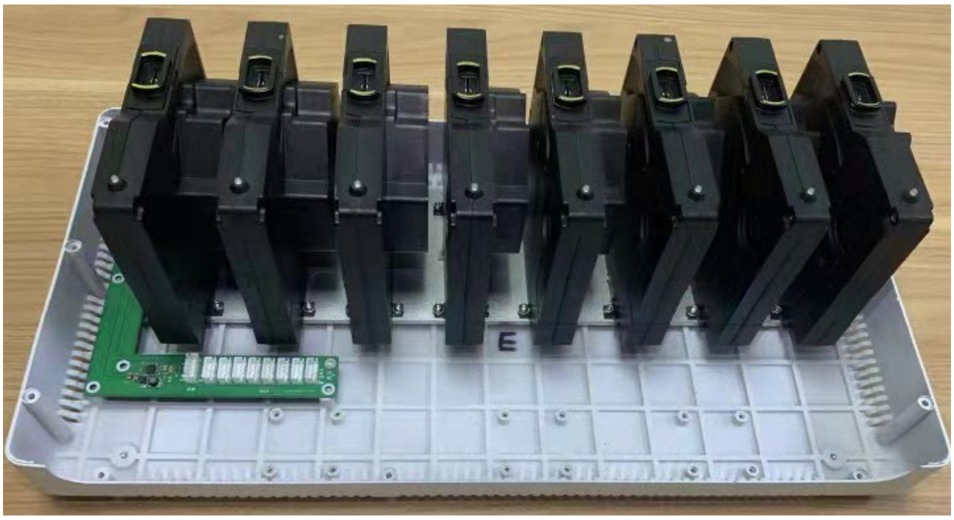

Supplementary Fig. S6. Correlation between image intensity and tumor percentage in gliomas. The tumor percentage for each sample was estimated by HE. Lengths of the 5 flower petals are qualitatively based on the correlation indexes and indicate the coefficients relative to the four circles representing -0.5, 0, 0.5 and 1. Blue petals represent areas of GradCAM; red petals represent T1wC; green petals represent T1w; orange petals represent T2w; pink petals represent FLAIR.

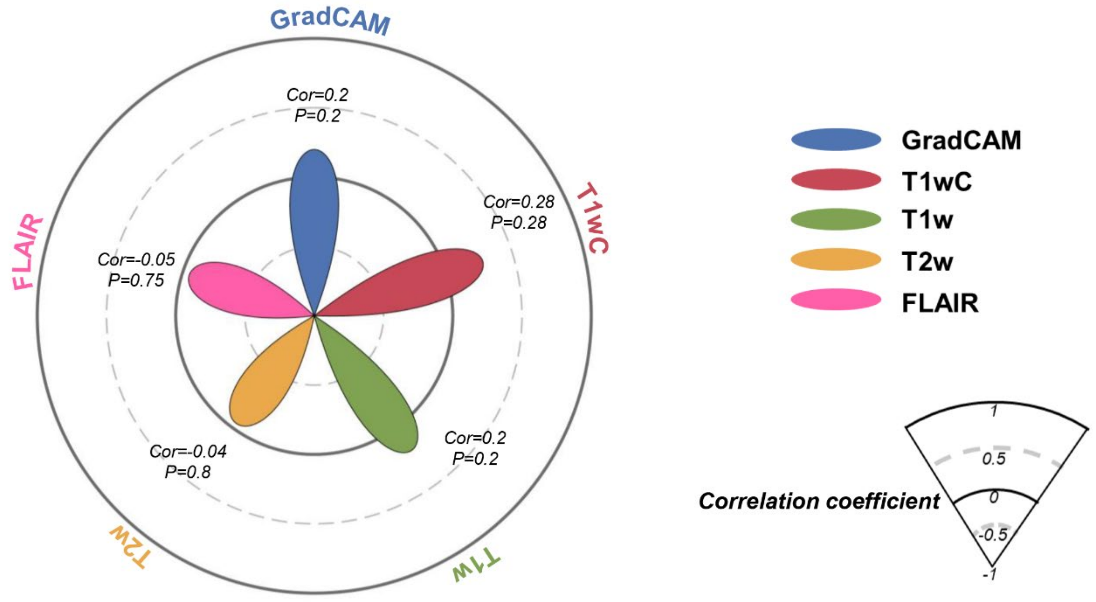

Abbreviations: GradCAM, Gradient-weighted Class Activation Mapping; T1wC, contrast-enhanced T1 weighted imaging; T1w, T1 weighted imaging; FLAIR, fluid-attenuated inversion recovery.

Supplementary Fig. S7. Correlation between image intensity and tumor purity in IDH-wt gliomas. Based on the data of WES, the tumor purity for each sample was identified by ABSOLUTE algorithm. Lengths of the 5 flower petals are qualitatively based on the correlation indexes and indicate the coefficients relative to the four circles representing -0.5, 0, 0.5 and 1. Blue petals represent areas of GradCAM; red petals represent T1wC; green petals represent T1w; orange petals represent T2w; pink petals represent FLAIR.

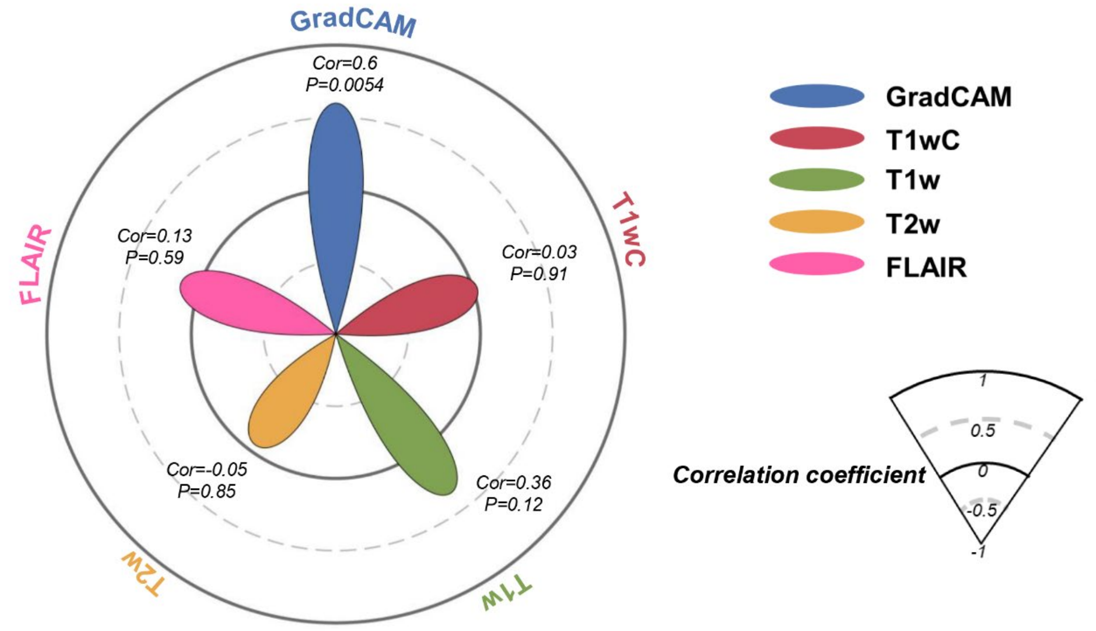

Abbreviations: GradCAM, Gradient-weighted Class Activation Mapping; T1wC, contrast-enhanced T1 weighted imaging; T1w, T1 weighted imaging; FLAIR, fluid-attenuated inversion recovery.

Supplementary Fig. S8. The summary of the distribution of variants in IDH-wildtype gliomas. (A) The types of the variants in gliomas. (B) The frequency of base substitutions within specific trinucleotide mutational contexts. (C) The classification of variants according to the functional effect. Most of the variants were missense mutations. (D) Top 10 genes with the highest counts of the variants.

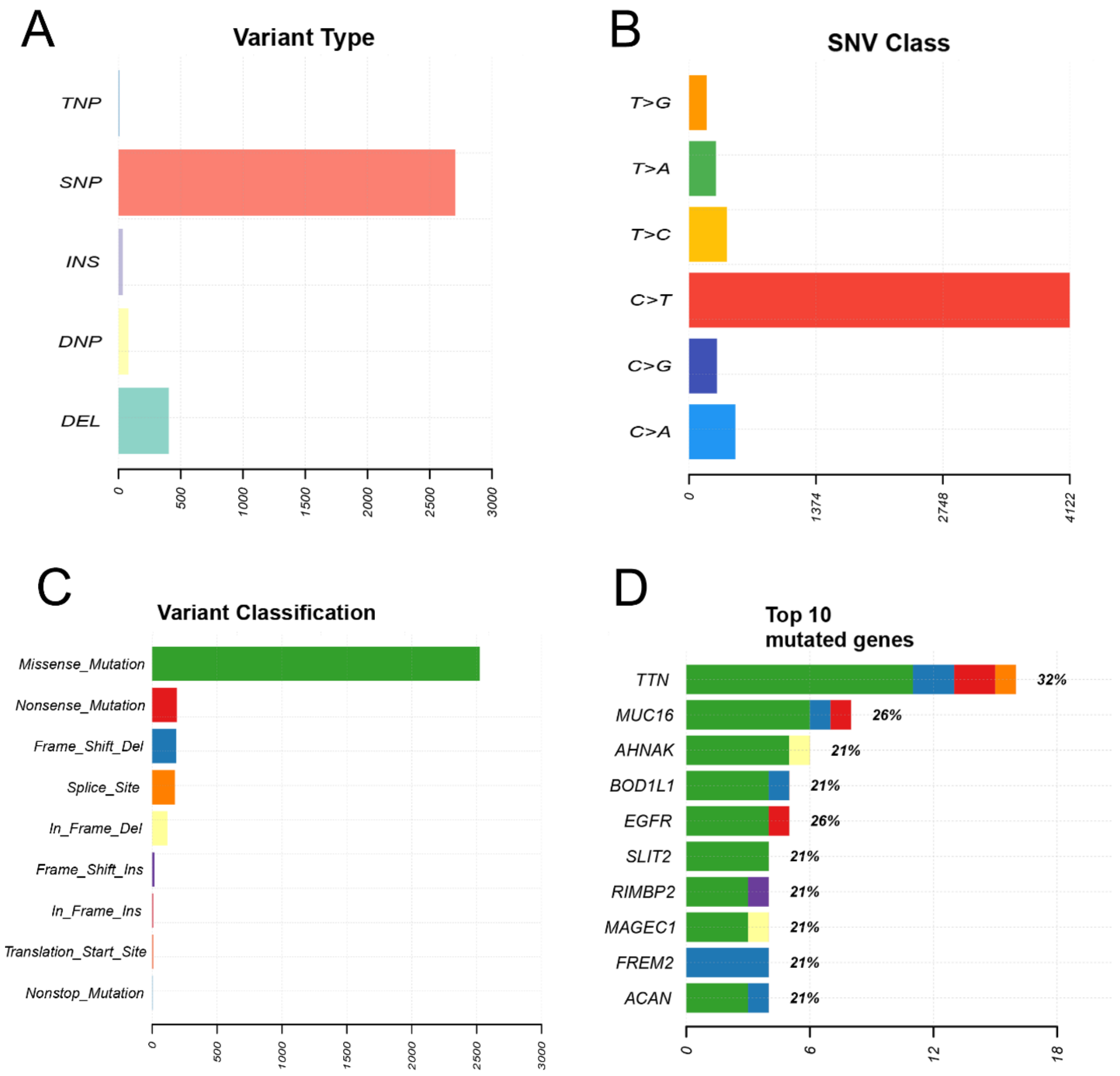

Abbreviations: TNP, trinucleotide polymorphism; SNP, single nucleotide polymorphism; INS, insertion: DNP, dinucleotide polymorphism; DEL, deletion; SNV, Single Nucleotide Variations.

Supplementary References

[1] M. Weller, M. van den Bent, M. Preusser, E. Le Rhun, J.C. Tonn, G. Minniti, M. Bendszus, C. Balana, O. Chinot, L. Dirven, P. French, M.E. Hegi, A.S. Jakola, M. Platten, P. Roth, R. Ruda, S. Short, M. Smits, M.J.B. Taphoorn, A. von Deimling, M. Westphal, R. Soffietti, G. Reifenberger, and W. Wick, EANO guidelines on the diagnosis and treatment of diffuse gliomas of adulthood. Nature Reviews Clinical Oncology 18 (2021) 170-186.

[2] M. Ceccarelli, F.P. Barthel, T.M. Malta, T.S. Sabedot, S.R. Salama, B.A. Murray, O. Morozova, Y. Newton, A. Radenbaugh, S.M. Pagnotta, S. Anjum, J. Wang, G. Manyam, P. Zoppoli, S. Ling, A.A. Rao, M. Grifford, A.D. Cherniack, H. Zhang, L. Poisson, C.G. Carlotti, Jr., D.P.d.C. Tirapelli, A. Rao, T. Mikkelsen, C.C. Lau, W.K.A. Yung, R. Rabadan, J. Huse, D.J. Brat, N.L. Lehman, J.S. Barnholtz-Sloan, S. Zheng, K. Hess, G. Rao, M. Meyerson, R. Beroukhim, L. Cooper, R. Akbani, M. Wrensch, D. Haussler, K.D. Aldape, P.W. Laird, D.H. Gutmann, H. Noushmehr, A. Iavarone, R.G.W. Verhaak, and T.R. Network, Molecular Profiling Reveals Biologically Discrete Subsets and Pathways of Progression in Diffuse Glioma. Cell 164 (2016) 550-563.

[3] K. Gorgolewski, C.D. Burns, C. Madison, D. Clark, Y.O. Halchenko, M.L. Waskom, and S.S. Ghosh, Nipype: a flexible, lightweight and extensible neuroimaging data processing framework in python. Frontiers in neuroinformatics 5 (2011) 13-13.

[4] T. Wolf, L. Debut, V. Sanh, J. Chaumond, C. Delangue, A. Moi, P. Cistac, T. Rault, R. Louf, M. Funtowicz, J. Davison, S. Shleifer, P. von Platen, C. Ma, Y. Jernite, J. Plu, C. Xu, T. Le Scao, S. Gugger, M. Drame, Q. Lhoest, A.M. Rush, and L. Assoc Computat, Transformers: State-of-the-Art Natural Language Processing, Conference on Empirical Methods in Natural Language Processing (EMNLP), Electr Network, 2020, pp. 38-45.

[5] A. Vaswani, N. Shazeer, N. Parmar, J. Uszkoreit, L. Jones, A.N. Gomez, L. Kaiser, and I. Polosukhin, Attention Is All You Need, 31st Annual Conference on Neural Information Processing Systems (NIPS), Long Beach, CA, 2017.

[6] K. He, X. Chen, S. Xie, Y. Li, P. Dollar, R. Girshick, and S.O.C. Ieee Comp, Masked Autoencoders Are Scalable Vision Learners, IEEE/CVF Conference on Computer Vision and Pattern Recognition (CVPR), New Orleans, LA, 2022, pp. 15979-15988.

[7] S.L. Carter, K. Cibulskis, E. Helman, A. McKenna, H. Shen, T. Zack, P.W. Laird, R.C. Onofrio, W. Winckler, B.A. Weir, R. Beroukhim, D. Pellman, D.A. Levine, E.S. Lander, M. Meyerson, and G. Getz, Absolute quantification of somatic DNA alterations in human cancer. Nat Biotechnol 30 (2012) 413-21.

[8] K. Tamura, G. Stecher, and S. Kumar, MEGA11 Molecular Evolutionary Genetics Analysis Version 11. Molecular Biology and Evolution 38 (2021) 3022-3027.
